# Development and validation of the NEOS2 score for prediction of long-term outcomes and improvement after first-line immunotherapy in patients with anti-NMDAR encephalitis: an international cohort study

**DOI:** 10.1016/j.lanepe.2025.101562

**Published:** 2025-12-11

**Authors:** Juliette Brenner, Anna E.M. Bastiaansen, Mar Guasp, Sergio Muñiz-Castrillo, Takahiro Iizuka, Marienke A.A.M. de Bruijn, Amaia Muñoz-Lopetegi, Eugenia Martínez-Hernández, Géraldine Picard, Alberto Vogrig, Mathilde Millot, Carsten Finke, Christian Geis, Jan Lewerenz, Nico Melzer, Harald Prüss, Saskia Räuber, Marius Ringelstein, Kevin Rostàsy, Kurt-Wolfram Sühs, Franziska S. Thaler, Klaus-Peter Wandinger, Katharina Wurdack, Yvette S. Crijnen, Jeroen Kerstens, Robin W. van Steenhoven, Sharon Veenbergen, Marco W.J. Schreurs, Robert van den Berg, Victor Volovici, Rinze F. Neuteboom, Juna M. de Vries, Peter A.E. Sillevis Smitt, Mariska M.P. Nagtzaam, Suzanne C. Franken, GENERATE Study Group, Dominica Ratuszny, Til Menge, Annikki Bertolini, Christian Bien, Robert Berger, Simone Tauber, Klemens Angstwurm, Thomas Seifert-Held, Andrea Kraft, Jaqueline Klausewitz, Ilya Ayzenberg, Katharina Eisenhut, Rosa Rößling, Martha Heiden, Tania Kümpfel, Josep Dalmau, Frank Leypoldt, Jérôme Honnorat, Maarten J. Titulaer

**Affiliations:** a Department of Neurology, Erasmus University Medical Center, Rotterdam, the Netherlands; b Department of Neurology, Hospital Clínic Barcelona - IDIBAPS, Spain; c French Reference Centre on Paraneoplastic Neurological Syndromes and Autoimmune Encephalitis, Hospices Civils de Lyon, Université Claude Bernard Lyon 1, Lyon, France; d Department of Neurology, Hospital Universitario 12 de Octubre, Madrid, Spain; e Department of Neurology, Kitasato University School of Medicine, Japan; f Department of Neurology, St. Elisabeth-Tweesteden Hospital, Tilburg, the Netherlands; g Department of Medicine (DMED), University of Udine, Italy; h Clinical Neurology, Department of Head-Neck and Neuroscience, Azienda Sanitaria Universitaria Friuli Centrale (ASUFC), Udine, Italy; i Department of Neurology, Charité Universitätsmedizin, Berlin, Germany; j Department of Neurology, Jena University Hospital, Germany; k Department of Neurology, University Hospital Ulm, Germany; l Department of Neurology, Medical Faculty and University Hospital Düsseldorf, Heinrich-Heine-University, Düsseldorf, Germany; m German Center for Neurodegenerative Diseases (DZNE), Berlin, Germany; n Center for Neurology and Neuropsychiatry, LVR-Klinikum, Heinrich-Heine-University Düsseldorf, Düsseldorf, Germany; o Department of Paediatric Neurology, Children's Hospital Witten/Herdecke University, Datteln, Germany; p Department of Neurology, Hannover Medical School, Germany; q Institute of Clinical Neuroimmunology, University Hospital and Biomedical Center, Ludwig-Maximilians-Universität, Munich, Germany; r Institute of Clinical Chemistry, University Hospital Schleswig-Holstein Kiel/Lübeck, Germany; s Department of Immunology, Erasmus University Medical Center, Rotterdam, the Netherlands; t Laboratory of Medical Microbiology and Immunology Microvida, Tilburg, the Netherlands; u Department of Neurosurgery, Erasmus University Medical Center, Rotterdam, the Netherlands; v CaixaResearch Institute, Barcelona, 08022, Spain; w Centro de Investigación Biomédica en Red, Enfermedades raras (CIBERER), Madrid 28029, Spain; x Department of Neurology, University Kiel, Germany

**Keywords:** Anti-NMDAR encephalitis, Prognosis, Outcome, Immunotherapy, Prediction score

## Abstract

**Background:**

Anti-N-methyl-d-aspartate receptor (anti-NMDAR) encephalitis is a severe disease that primarily affects young people and can improve with adequate treatment. We aimed to refine the anti-NMDAR Encephalitis One-year functional Status (NEOS) score by developing NEOS2, an updated model using readily available data at the time of diagnosis. We assessed the predictive value of the NEOS2-score for (1) improvement following first-line treatment, (2) functional outcome at one-year follow-up, and (3) resumption of school or work within three years.

**Methods:**

In this international (France, Germany, Japan, the Netherlands and Spain) cohort study in patients with a definite anti-NMDAR encephalitis diagnosis (according to the clinical criteria plus antibody testing in CSF), we performed logistic regression analyses to develop and validate multivariable models to predict -based upon variables available at diagnosis- short (ΔmRS two weeks after first-line treatment), middle (modified Rankin Scale [mRS] at one year), and long-term (return to school or work within three years) outcomes. We included clinical variables and biomarkers available at diagnosis.

**Findings:**

We included 702 patients (mean age 23 years, 95%-CI 2–69; 79% female, 21% male) diagnosed between the discovery of the disease in 2007 and 2022. Most patients (96%; 672/702) had received first-line immunotherapy, and 38% (233/615) showed improvement within two weeks. One year after diagnosis, 80% (517/644) had a favourable functional outcome (mRS≤2). At three years, 73% (203/278) had resumed work/school. In multivariable analysis, higher age (odds ratio [OR] 0·35, 95%-CI 0·29–0·43, p < 0·0001), treatment delay (OR 0·49, 95%-CI 0·41–0·58, p < 0·0001), movement disorders (OR 0·32, 95%-CI 0·24–0·41, p < 0·0001), ICU-requirement (OR 0·34, 95%-CI 0·26–0·44, p < 0·0001) and increased CSF leucocyte count (OR 0·65, 95%-CI 0·60–0·71, p < 0·0001) independently predicted poorer outcomes (NEOS2, accuracy AUC 80%, 95%-CI 75–86%). The same variables, excluding age, were relevant in predicting improvement following first-line immunotherapy (NEOS2-T AUC 81–84%, 95%-CI 77–86%). Return-to-work or -school served as a useful measure of longer-term outcomes, predicted with equal accuracy as one-year functional outcome (NEOS2-W AUC 80%, 95%-CI 75–85%). The NEOS2-score, applied as an ordinal measure, enabled nuanced predictions of outcome probabilities across the score spectrum, ranging from a high (80%; n = 20/25) likelihood of improving after first-line immunotherapy and achieving a good outcome (100%; n = 32/32) to a high risk of first-line treatment failure (97%; n = 77/79) and no return to school/work (94%; n = 15/16).

**Interpretation:**

The NEOS2-score, readily available at diagnosis and easy to apply, can identify patients with either a favourable or poor prognosis, and those who may benefit from early intensified treatment. The value of the NEOS2-score for guiding treatment decisions and as a stratification tool in studies on optimal treatment regimens, should be confirmed in further prospective studies.

**Funding:**

This study was funded by 10.13039/501100010573Dioraphte (charity; project 2001 0403).

**Research in context**

**Evidence before this study**

We have searched PubMed, Google Scholar and key references from database inception up to 31 December 2023 for investigated biomarkers predicting outcomes of anti-NMDAR encephalitis (MESH terms for biomarker, prognosis, outcome, autoimmune encephalitis, Anti-N-Methyl-d-Aspartate Receptor encephalitis), prioritising factors accessible in daily clinical practice and evident at diagnosis. The negative impact of increasing age has been largely elucidated. Clinical disease severity (mRS, need for ICU admission), immunological activity (number of leukocytes in CSF), treatment delay, and (lack of) response to first-line immunotherapy have been independently related to outcome, as well as demonstrated to have an additive prognostic value, captured by the ‘anti-NMDAR Encephalitis One-year functional Status (NEOS)' score. Radiological involvement of the hippocampus has been associated with persistent cognitive deficits, and serum and CSF markers for inflammation (i.e. CXCL13) or neuronal damage (i.e. NfL) have also been investigated as biomarkers for outcome. Treating physicians are trying to predict prognosis and determine the best treatment strategy in the often young and severely affected anti-NMDAR encephalitis patients. The NEOS score has proven relevant for predicting outcomes in anti-NMDAR encephalitis and is widely applied. A lack of response to first-line treatment is a pertinent reason to escalate treatment, currently the most applied strategy in treating anti-NMDAR encephalitis. This is incorporated in the NEOS score, which included ‘(lack of) improvement within four weeks of treatment with first-line immunotherapy’ as a predictive variable for poor outcomes. It would be a significant step forward to identify patients at risk for a poor (long-term) outcome and/or for failure of first-line therapy, directly at diagnosis. This allows for early treatment escalation if required, instead of awaiting the effect of first-line treatment for weeks.

**Added value of this study**

The NEOS2 model does not necessitate response to first-line treatment as a predictive variable and can predict outcomes of anti-NMDAR encephalitis with equal accuracy as the original NEOS model. Additionally, the very good performance of the NEOS2-T model, predicting response to first-line treatment with a similar set of predictive variables, proves that the patients at risk for a poor outcome are often the same patients at risk for failure of first-line treatment; these are the patients potentially benefitting from early intensified and maybe more aggressive treatments.

A limitation of the original NEOS score is the use of functional independence (expressed on the mRS) as the main outcome measure. As the majority of patients become independent within one or two years after diagnosis and treatment of the anti-NMDAR encephalitis, this outcome is largely skewed, limiting the power of prognosticating longer-term outcomes. We demonstrate that the ability to return to work or school can be used as a substitute outcome measure for the (very) long-term, when the majority of patients has returned to functional independence, and can be predicted with equal accuracy. Applying more demanding outcome measures, like return to work/school, is more relevant to patients and allows to differentiate within patients assigned a ‘favourable outcome’ by generic outcome measures.

**Implications of all the available evidence**

The NEOS2, NEOS2-W, and NEOS2-T models are easily applicable in daily clinical practice and can accurately identify patients at risk for a poor functional outcome, who may benefit from early intensified treatment. The use of the NEOS2-score to stratify patients within trials would probably increase the power and reduce the needed sample size.

## Introduction

Anti-N-methyl-d-aspartate receptor (anti-NMDAR) encephalitis is a severe but treatable disease.^1^^,^^2^ It is one of the most prevalent types of autoimmune encephalitis, which incidence has increased due to enhanced awareness and recognition of the disorder.^3^^,^^4^ Despite being potentially reversible with prompt immunotherapy,^1^ the disorder can be lethal and frequently results in persisting sequelae. Although functional neurological outcomes are good –around 80% of patients are independent after a year–, a similar proportion of patients continue exhibiting persistent cognitive and neuropsychiatric deficits.^1^^,^^5^, ^6^, ^7^, ^8^ A third of patients does not return to work or school, and additional patients need adjustments.^5^^,^^8^^,^^9^ This is especially relevant as most anti-NMDAR encephalitis patients are young.

The prognosis of anti-NMDAR encephalitis has been associated to several person- and disease-related factors. The probability of a good outcome decreases with age,^3^^,^^10^ and younger children also have a poorer outcome.^1^^,^^11^ A preceding herpes simplex virus (HSV) Encephalitis and radiological involvement of the hippocampus are associated with persistent cognitive deficits.^6^^,^^8^^,^^12^ Clinical disease severity, immunological activity (increased leucocyte-count in cerebrospinal fluid [CSF], antibody titres),^13^ treatment delay, and lack of response to first-line immunotherapy have all been related to outcome.^1^^,^^14^

The multivariable anti-NMDAR Encephalitis One-year functional Status (NEOS) score offers moderate but meaningful predictive power for outcomes (on the modified Rankin Scale [mRS]^15^) one year after onset of the encephalitis.^14^ An important limitation of the NEOS-score is the variable ‘improvement within four weeks following the start of first-line immunotherapy’. This means the score can only be applied one month after diagnosis and treatment, limiting its informativeness and its value in guiding initial therapeutic decisions.^14^ This study addresses this limitation by developing an updated model capable of predicting outcomes based on clinical information available at the time of diagnosis (‘NEOS2’). In addition, we introduce two novel extensions as (1) we investigated prediction of early improvement following first-line immunotherapy (‘NEOS2-T’) and (2) we included the likelihood of returning of returning to work or school within three years of disease onset as a sensitive outcome metric for the longer-term (‘NEOS2-W’), when most patients have returned to independent daily living. Estimating the likelihood of response to first-line immunotherapy (NEOS2-T) –in addition to (longer-term) outcome– would facilitate the early identification of patients who may require more aggressive treatment. For patients with a good prognosis after first-line immunotherapy, potentially harmful side-effects of costly second-line treatments could be avoided. An early prediction score could also support patient stratification in clinical trials, potentially enhancing statistical power in studies with limited sample sizes, as anti-NMDAR encephalitis is a rare disease.

The introduction of sensitive longer-term outcome metrics (NEOS2-W) allowed us to explore whether the factors contributing to one-year functional outcomes also relate to longer-term, patient-relevant endpoints (NEOS2-W), supporting their sustained relevance and the value of one-year functional outcome as an actionable surrogate to guide treatment strategies at diagnosis.

Therefore, the primary objective of the current study was to develop a ‘NEOS2’ prediction score providing relevant information for short (NEOS2-T), middle (NEOS2), and long (NEOS2-W) term outcomes based on clinical data available at diagnosis.

## Methods

### Study design and participants

In this international cohort study, we combined the data from five national anti-NMDAR encephalitis cohorts (France, Germany, Japan, the Netherlands, and Spain) to develop prediction models. All patients were diagnosed between the discovery of the disease in 2007 and 2022, met the criteria for definite anti-NMDAR encephalitis, tested positive for antibodies in CSF, and had at least four months of follow-up data available (or died <4 months; 5% had <12 months but >4 months of follow-up).^16^ Patients were excluded if (1) their premorbid mRS or Paediatric Cerebral Performance Category (PCPC) scores^17^ were over two, preventing them from attaining positive primary outcomes (low mRS/PCPC), or (2) the anti-NMDAR encephalitis was preceded by HSV encephalitis, potentially influencing clinical parameters and outcomes.

Two of the larger cohorts, from the Netherlands and France, represent unbiased reflections of the disease population, providing national coverage. As the clinical characteristics between the national cohorts differed (Supplementary Table S1; Supplementary Material), we added ‘cohort’ as an independent variable in the analyses.

### Ethics approval

This observational cohort study was reviewed by the Institutional Review Board of the Erasmus MC (MEC-2020-0418; date 22-06-2020) and found not to require WMO (Dutch Medical Research Involving Human Subjects Act)-governed ethics approval. All patients in the Netherlands provided written informed consent within this study before inclusion. Written informed consent was obtained from all patients in the international cohort upon enrolment in the respective cohorts (France, Germany, Japan, Spain).

### Definition of variables and handling missing data

Potential predictive variables were selected based on a literature review and their availability at diagnosis. Data on 20 predictive variables and seven outcome variables (three main outcomes, represented binary, and time to achieve these outcomes) were collected for every cohort (Supplementary Data Dictionary). The primary outcome variables were ‘functional status one year after diagnosis (mRS/PCPC)’ and ‘early improvement following first-line treatment’. Functional status was defined by mRS or PCPC scale and dichotomised (1 for a favourable functional outcome, 0 for a poor outcome), defining as ‘favourable outcome’ a score ≤2 (independence in mobility and daily activities, or at an age-appropriate level). Improvement following first-line immunotherapy was defined as an improvement of at least one point on the mRS (ΔmRS≥1), obtained two weeks after administering treatment. Return-to-work/school was also included as a (secondary) binary outcome, supported by the time to return to work/school. The majority (96%; 672/702) of included patients had received first-line immunotherapy; only these patients were included in the model assessing improvement following treatment. Patients who did not attend school or work before the illness, were excluded from the return-to-work/-school analysis.

Data quality assessments were conducted, with source verification and corrections made as needed. Skewed variables were transformed (Supplementary Data Dictionary); reported values are geometric descriptive statistics. Within predictive and primary outcome variables, 3% of the values were missing; no variable contained over 15% missing data. Missing values occurred completely at random and were imputed with five iterations, using the MICE package in R.e1 The original and five imputed datasets were randomly split into 70% for development of the multivariable prediction models and 30% for validation, ensuring equal distributions of cohort and the two primary outcomes (Appendix A, Appendix A).

### Statistical analysis - univariable analysis

All Statistical analyses were performed in R (v4·3·2; R Core-Team 2023).e2 Univariable analyses were performed on the original data. For comparisons of cohorts (international variability, development versus validation) and groups based on outcome (univariable analysis), we have applied Pearson's Chi Squared tests in case of binary variables, Wilcoxon rank sum and Kruskall–Wallis tests for ordinal variables, and T-tests or ANOVA's for continuous variables. For the univariable analysis of potential predictive continuous variables, effects were additionally visualised and fitted with local polynomial regression curves to check for linearity. Linear effects were analysed with t-tests, while univariable logistic regression with polynomials or restricted cubic splines (depending on the curve) was applied for non-linear effects. To explore the magnitude of the influence certain predictive variables can have on outcomes, we extended the analyses with preliminary time-to-event analyses, with time to reach the primary and secondary outcomes as the dependent variables. In these analyses, death and relapse were treated as competing risks because they prevent the occurrence of the main outcome. Patients who were retired or unemployed before the encephalitis were excluded from the time-to-return-to-work/school analysis. If an endpoint was not reached, cases were censored at the time of last follow-up.

### Statistical analysis - multivariable regression analysis

All potential predictive variables were considered in the multivariable analysis to allow the identification of additive or opposite effects. Nested hierarchical mixed-effects logistic regression models were fitted to the development-parts of the five imputed datasets for the primary outcomes. A random intercept for cohort was included to control for potential international differences (Supplementary Material). We controlled for multicollinearity by determining correlations between independent variables (Supplementary Figure S1; no correlations>0·7) and variance inflation factors (no VIFs>5). Interactions between predictive variables were tested and included where relevant. Variables were selected through backwards stepwise selection based on the pooled sampling variance across the five imputed datasets and the Akaike Information Criterion (AIC). We used the psfmi package for this.e3 Pooled sampling variance was calculated using Rubin's rules to account for the uncertainty introduced by multiple imputation. The AIC was used to balance model fit and complexity. Estimates and p-values of the model in the different imputed datasets were also pooled. Performance of the models was assessed in the original development and validation datasets by evaluating (1) discriminative ability, determined by plotting receiver operating characteristic (ROC) curves and calculating the area under the curve (AUC), sensitivity and specificity of the model, (2) model calibration, and (3) the Goodness-of-Fit test of Hosmer&Lemeshow.

### Statistical analysis - developing the NEOS2-models

To build the NEOS2-scores, natural numbers were assigned to categories of the predictive variables based on effect sizes/odds ratio's (OR). For continuous variables, categories were defined based on the distribution of the data (quartiles) and reference values (i.e. <6 × 10^6^/L leukocytes in CSF), size (OR) and direction (splines) of the effect. Non-linear/exponential effects were incorporated in the magnitude of the numbers. Interaction effects were captured by assigning numbers to composite categories, based on model calculations. The categories in the final model account for 14 degrees of freedom, not exceeding the limit of 10% of cases with a particular outcome, minimising the risk of overfitting.

### Role of the funding source

The funder of the study had no role in study design, data collection, data analysis, data interpretation or writing of the report.

## Results

### Clinical characteristics

A total of 702 anti-NMDAR encephalitis patients from France (n = 324), Germany (n = 170), Japan (n = 45), the Netherlands (n = 132), and Spain (n = 31) were included. The international cohort reflected the characteristics of the patient population (Table 1; 79% female; average age at disease onset 23 years, standard deviation [SD] 2 years, 95%-CI 2–69 years). Two-hundred thirty-three patients (38%) improved (ΔmRS≥1) within two weeks of first-line immunotherapy (‘early improvement’); improving patients were less likely to receive second-line therapy (46% vs 79% of patients without improvement). Functional outcomes after one year were considered favourable (mRS/PCPC≤2) in 517/644 patients (80%). Recovery continued beyond twelve months after diagnosis; in the average follow-up of 30 months (SD 2, range 4–366 months), 580/647 patients (90%) reached functional independence (mRS/PCPC≤2).

**Table 1:** Baseline characteristics.

	All included patients, N = 702
Baseline characteristics	
Sex^a^	
Female, n/N (%)	557/701 (79)
Male, n/N (%)	144/701 (21)
Age at disease onset, Median (IQR, Min.-Max.)	22 (16–31, 1–87)
Paediatric patients (<18 years at disease onset), n/N (%)	202/702 (29)
Ethnicity^a^	
Caucasian, n/N (%)	450/646 (70)
Asian, n/N (%)	80/646 (12)
Black/African/Caribbean, n/N (%)	77/646 (12)
Latin/Hispanic, n/N (%)	7/646 (1)
Other, n/N (%)	32/646 (5)
Follow-up time (months), Median (IQR, Min.-Max.)	30 (14–57, 0–366)
Patients with ≥1 year of follow-up data, n/N (%)	670/702 (95)
Patients with ≥3 years of follow-up data, n/N (%)	278/702 (40)
Clinical variables	
Tumour, n/N (%)	192/682 (28)
Altered consciousness at presentation, n/N (%)	383/697 (55)
Seizures, n/N (%)	512/696 (74)
Cognitive disturbances, n/N (%)	628/693 (91)
Behavioural changes, n/N (%)	649/693 (94)
Language disorders, n/N (%)	457/695 (66)
Movement disorders, n/N (%)	430/699 (62)
Autonomic dysregulation, n/N (%)	308/689 (45)
Sleep disorders, n/N (%)	281/663 (42)
Severity (mRS) at diagnosis, Median (IQR, Min.-Max.)	3 (3–5, 1–5)
ICU admission, n/N (%)	382/697 (55)
Ancillary tests	
MRI abnormalities, n/N (%)	205/643 (32)
EEG abnormalities, n/N (%)	541/611 (89)
Abnormalities in posterior rhythm, n/N (%)	332/594 (56)
CSF leucocyte count (x10^6^/L), Median (IQR, Min.-Max.)	22 (8–60, 0–848)
CSF antibody titre (1 in …), Median (IQR, Min.-Max.)	32 (10–100, 1–10240)
Diagnosis & Treatment	
Diagnostic delay (weeks), Median (IQR, Min.-Max.)	4 (2–9, 1–835)
Treatment delay (weeks), Median (IQR, Min.-Max.)	3 (1–6, 0–229)
Received first-line immunotherapy, n/N (%)	672/702 (96)
Intravenous methylprednisolone (IVMP), n/N (%)	595/701 (85)
Intravenous immunoglobulins (IVIG), n/N (%)	548/699 (78)
Plasmaphaeresis, n/N (%)	218/699 (31)
Second-line therapy, n/N (%)	429/701 (61)
Improvement two weeks after first-line therapy (at least one point on the mRS), n/N (%)	233/615 (38)

	Follow-up data ≥ 1 year, N = 670

Long-term outcome	
Outcome (mRS) twelve months after diagnosis, Median (IQR, Min.-Max.)	1 (0–2, 0–6)
Patients with a good outcome (mRS≤2) after twelve months, n/N (%)	517/644 (80)
Reached independence (mRS≤2) during follow-up, n/N (%)	580/647 (90)
Time to independence (months), Median (IQR, Min.-Max.)	4 (2–7, 1–48)
Ability to go back to work/school, n/N (%)	442/594 (74)
Time to return to work/school (months), Median (IQR, Min.-Max.)	9 (4–16, 0–92)
Back at work at three years after diagnosis, n/N (%)	203/278 (73)
Relapse, n/N (%)	101/635 (16)
Time to relapse (months), Median (IQR, Min.-Max.)	18 (8–44, 3–366)

Abbreviations: mRS = modified Rankin Scale, CSF = cerebrospinal fluid, n = number of participants with the feature, N = total number of participants with data, IQR = interquartile range, Min. = minimum, Max. = maximum.

a Self-reported.

The development (n = 494) and validation (n = 208) datasets were equivalent in terms of clinical severity and outcomes (Supplementary Tables S2 and S3).

### Prognosticating functional status

Many variables were (univariably) associated with functional outcome one year after diagnosis (Supplementary Table S4). The effect of age was non-linear; paediatric patients under the age of twelve had slightly poorer outcomes than individuals aged twelve to 36 years, and the likelihood of a favourable outcome decreased exponentially with age in adults older than 36 years. Timing of the (diagnostic) lumbar puncture (in relation to symptom onset), and treatment delay/initiation (after symptom onset) were highly correlated (Pearson's R = 0·84); to avoid multicollinearity, we only included treatment delay. In multivariable analysis, age (with splines; OR >36 years 0·35, 95%-CI 0·29–0·43, p < 0·0001), treatment delay (linear; OR 0·49, 95%-CI 0·41–0·58, p < 0·0001), movement disorders (OR 0·32, 95%-CI 0·24–0·41, p < 0·0001), ICU admission (OR 0·34, 95%-CI 0·26–0·44, p < 0·0001), and a higher CSF leucocyte count (OR 0·65, 95%-CI 0·60–0·71, p < 0·0001) remained as independent predictors for a decreased likelihood of good outcome (Table 2). A significant interaction of leucocyte count in diagnostic CSF and treatment delay suggested a more pronounced effect of a leucocyte increase when the lumbar puncture (often directly followed by commencing treatment) was performed early after symptom onset (Supplementary Figure S2).

**Table 2:** Multivariable analysis of predictors for functional outcome after one year, improvement after first-line therapy, and returning to work or school.

	NEOS2			NEOS2-T			NEOS2-W		
	Estimate (β) (standard error)	Odds ratio (OR) (95%-CI)	p-value	Estimate (β) (standard error)	Odds ratio (OR) (95%-CI)	p-value	Estimate (β) (standard error)	Odds ratio (OR) (95%-CI)	p-value
**Predictive variable**									
Intercept	4·83 (0·35)		<0·0001∗	1·77 (0·15)		<0·0001∗	3·90 (1·11)		0·00046∗
Age disease onset^a^									
First spline (0–12 years)	0·29 (0·13)	1·35 (1·04–1·73)	0·020∗				0·26 (0·33)	1·30 (0·68–2·49)	0·42
Second spline (13–36 years)	0·032 (0·083)	1·03 (0·88–1·22)	0·70				−0·40 (0·20)	0·67 (0·45–0·98)	0·039∗
Third spline (>36 years)	−1·04 (0·097)	0·35 (0·29–0·43)	<0·0001∗				−1·57 (0·33)	0·21 (0·10–0·38)	<0·0001∗
Movement disorders	−1·15 (0·14)	0·32 (0·24–0·41)	<0·0001∗	−1·02 (0·075)	0·36 (0·31–0·42)	<0·0001∗	−0·58 (0·30)	0·56 (0·31–0·96)	0·042∗
No movement disorder	0	1		0	1		0	1	
Required ICU admission	−1·08 (0·13)	0·34 (0·26–0·44)	<0·0001∗	−1·15 (0·076)	0·32 (0·27–0·37)	<0·0001∗	−0·84 (0·31)	0·43 (0·23–0·78)	0·0063∗
Not admitted to the ICU	0	1		0	1		0	1	
CSF leucocyte count (x10^6^/L)^b^	−0·43 (0·041)	0·65 (0·60–0·71)	<0·0001∗	−0·27 (0·027)	0·76 (0·72–0·80)	<0·0001∗	−0·30 (0·094)	0·74 (0·62–0·89)	0·0017∗
Treatment delay (weeks)^b^	−0·72 (0·092)	0·49 (0·41–0·58)	<0·0001∗	−0·12 (0·055)	0·89 (0·80–0·98)	0·033∗	−0·82 (0·22)	0·44 (0·28–0·68)	0·00025∗
Interaction (treatment) delay x leukocytes	0·13 (0·016)	1·14 (1·10–1·18)	<0·0001∗	0·049 (0·012)	1·05 (1·03–1·07)	<0·0001∗	0·13 (0·040)	1·13 (1·05–1·23)	0·0015∗

Outcome variable	Independent (mRS≤2) one year after diagnosis			Improvement two weeks after first-line treatment			Back at work/school after three years		
Model performance	Hosmer&Lemeshow X^2^ = 3·18 (p = 0·92)			Hosmer&Lemeshow X^2^ = 10·71 (p = 0.22)			Hosmer&Lemeshow X^2^ = 2·60 (p = 0·96)		

Estimates and corresponding odds ratios of the variables persisting in the multivariable analyses for predicting (1) good functional outcome one year after diagnosis (mRS≤2; NEOS2), (2) early (≤2 weeks) improvement (ΔmRS≥1) following first-line treatment (NEOS2-T), and (3) return to work/school within three years after diagnosis (NEOS2-W). Except for age being irrelevant for early improvement following treatment, the same variables persisted in all three multivariable analyses. The interaction-term for number of leukocytes in diagnostic CSF and time to treatment initiation caused an attenuating effect of treatment delay (often coinciding with delay to the diagnostic lumbar puncture) on the negative impact of a high leucocyte number in CSF. Abbreviations: NEOS2 = the Second anti-NMDAR Encephalitis One-year functional Status score, NEOS2-T = the anti-NMDAR Encephalitis Outcome Score after Treatment, NEOS2-W = the anti-NMDAR Encephalitis Outcome Score for Work or education, ICU = intensive care unit, CSF = cerebrospinal fluid, CI = confidence interval.

The corresponding univariable analyses are provided in Appendix A, Appendix A, Appendix A.

∗p < 0.05.

a Square root to transform.

b Log2-transformed.

**Fig. 1:**
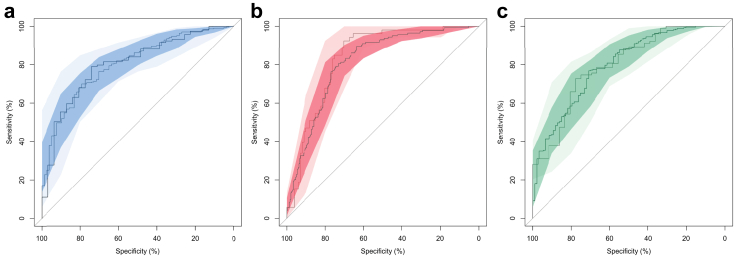
**ROC curves of the NEOS2, NEOS2-T and NEOS2-W models**. A: Receiver Operator Characteristics (ROC) of the multivariable model for functional outcome (independence) one year after diagnosis of anti-NMDAR encephalitis, showing very good discriminative ability (AUC 80% in the development [n = 388] and validation [n = 150] dataset, 95%-CI 75–86% and 72–89%) and good specificity (79% in the development dataset, 95%-CI 70–87%, 74% in validation, 95%-CI 62–85%), and sensitivity (70% in the development dataset, 95%-CI 65–75%, 79% in validation, 95%-CI 70–85%). B: ROC of the multivariable model for early improvement after first-line treatment of anti-NMDAR encephalitis, showing very good discriminative ability (AUC 81 and 84% in the development [n = 383] and validation [n = 154] dataset respectively, 95%-CI 77–86% and 78–90%) and sensitivity (81 and 93% respectively, 95%-CI 73–86% and 82–97%) and good specificity (73% and 70%, 95%-CI 67–78% and 95%-CI 61–78%). C: ROC of the multivariable model for likelihood to return-to-work/school three years after diagnosis of anti-NMDAR encephalitis, showing very good discriminative ability (80% in the development [n = 370] and validation [n = 139] dataset, 95%-CI respectively 75–85% and 72–88%), and good sensitivity (77% in the development dataset, 95%-CI 72–81%, 73% in validation, 95%-CI 64–80%) and specificity (71%, 95%-CI 61–79%, and 78%, 95%-CI 62–88%). In all figures the darker line/area represents the characteristics in the original development dataset and the lighter shade the characteristics in the validation dataset, indicating a good consistency of the models across datasets.

Fitting the multivariable model to the five imputed datasets resulted in the same selection of variables and similar performance of the models across imputations (Supplementary Figure S3). The random intercept for cohort was dropped in the multivariable analyses, suggesting that the international variability in disease severity and outcome was adequately explained by the relation between predictive variables and outcome variables in the model. This was confirmed by the good performance of the model across all five cohorts (Supplementary Figure S3). The multivariable logistic regression model had good discriminative ability (AUC 80% in the development and validation dataset, 95%-CI 75–86% and 72–89% respectively), specificity (79% in the development dataset, 95%-CI 70–87%, 74% in validation, 95%-CI 62–85%) and sensitivity (70% in the development dataset, 95%-CI 65–75%, 79% in validation, 95%-CI 70–85%) for predicting independence one year after diagnosis of anti-NMDAR encephalitis (Fig. 1A). The model was well-calibrated (Supplementary Figure S4) and fitted the data well (Hosmer&Lemeshow X^2^ = 3·18, p = 0·92). The model performance was equal to that of the original NEOS-model (AUC 80%; Supplementary Figure S5) and significantly better than the performance of a modification of the NEOS-model excluding the variable on improvement after first-line therapy (AUC 68%; Supplementary Figure S6).

### Prognosticating improvement following first-line immunotherapy

Except for age, the same predictive variables as in the model for outcome were relevant for predicting early improvement following first-line therapy (Table 2; Supplementary Table S5). The multivariable logistic regression model for predicting improvement following first-line immunotherapy had good discriminative ability (AUC 81–84% in the development and validation dataset respectively, 95%-CI 77–86% and 78–90%), sensitivity (81–93% respectively, 95%-CI 73–86% and 82–97%) and specificity (73 and 70% respectively, 95%-CI 67–78% and 61–78%) (Fig. 1B). The model was well-calibrated (Supplementary Figure S7) and fitted the data well (Hosmer&Lemeshow X^2^ = 10·71, p = 0·22).

### Prognosticating return-to-work/-school

As 90% of patients became independent (mRS/PCPC≤2) over time, a more discriminative and demanding outcome measure was considered for the longer-term: return-to-work or -school. One year after diagnosis, 47% of all patients had resumed work/school (274/583), increasing to 73% of patients at three years (203/278). Of all patients returning-to-work/school (450/620; 73%), 64% did so within the first year (274/428) and 97% (416/428) within three years after diagnosis. It took patients on average 8 months (SD 1, 95%-CI 0–44) after diagnosis to resume work/school. The probability of resuming work/school within 36 months could be predicted with the same variables and with equal accuracy (AUC 80% in the development and validation dataset, 95%-CI respectively 75–85% and 82–88%; sensitivity 77% in the development dataset, 95%-CI 72–81%, 73% in validation, 95%-CI 64–80%; specificity 71%, 95%-CI 61–79%, and 78%, 95%-CI 62–88%, respectively; Fig. 1C) as the primary outcomes (Table 2; Supplementary Table S6).

### Developing the NEOS2-models

**Fig. 2:**
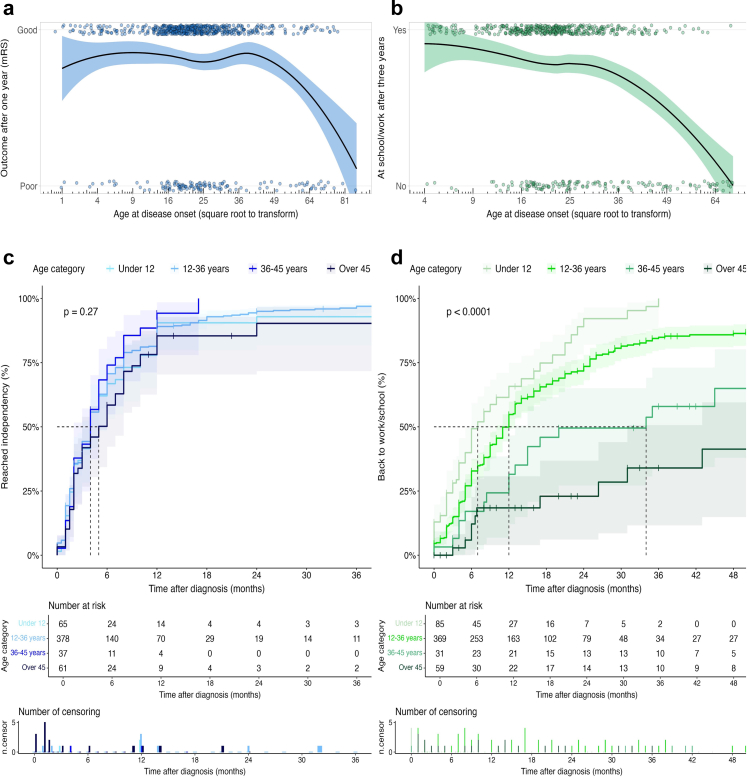
**Effect of age on the probability and timing of returning to independence and returning to work or school**. A: The relation between age and outcome is non-linear, plotted here (A & B) with restricted cubic splines at 12 and 36 years. Paediatric patients under 12 years (first dotted line) had on average slightly poorer outcomes (79%) than patients aged 12–36 years (82%), and the likelihood of a good functional outcome exponentially decreased with age after 36 years (70% at 45 years, 50% above 60 years) B: Although the effect of age on the likelihood to return to work or school also exponentially increases after 30–36 years (68% of patients above the age of 36 years reintegrate, 39% over 45 years and 5% [2/37 patients] over the age of 60 years), this effect is unidirectional and young children (<12) have the highest likelihood to return to school (85%). C & D: Time-to-event curves for reaching independence and returning to work/school, categorised by age. Return to work/school is a more discriminative item to assess long-term functional outcome than independence, showing both an incrementally smaller proportion of patients and a longer duration to return to school/work with increasing age.

Natural numbers were attributed to the categories of the predictive variables according to their calculated odds within the context of the complete model (Table 3; Supplementary Method). Categories for continuous predictive variables were defined based on the distribution of the data (quartiles; Appendix A, Appendix A, Appendix A), reference values and directionality of the relation to the outcomes (i.e. linear, polynomial, splines). For example for age, categories were defined at <12 years (highest likelihood to return to work/school within three years, not necessarily favourable for functional outcome after one year), 12–36 years (best functional outcome), and three categories >36 years to incorporate the increasing effect of age beyond 36 years (Fig. 2).

**Table 3:** NEOS2, NEOS2-T and NEOS2-W score.

Predictive variable	NEOS2	NEOS2-T	NEOS2-W
Age disease onset			
0–12 years	1	–	0
13–36 years	0	–	1
37–45 years	1	–	3
46–60 years	2	–	6
> 60 years	4	–	9
Movement disorder			
No	0	0	0
Yes	2	2	1
Requires ICU admission			
No	0	0	0
Yes	2	2	2
CSF leukocytes (x10^6^/L)			
Not elevated (<6)	0	0	0
6–50			
Timing within 2 weeks after onset	2	2	2
Timing after 2 weeks	1	1	1
> 50			
Timing within 2 weeks after onset	4	4	4
Timing after 2 weeks	2	2	2
Treatment delay			
< 2 weeks	0	0	0
2 weeks–1 month	1	1	1
1–2 months	2	1	2
> 2 months	4	2	4

**Maximum total score**	16	10	20

Natural numbers assigned to categories of the predictive variables in the models for (1) functional outcome one year after diagnosis (mRS≤2; NEOS2), (2) early (≤2 weeks) improvement (ΔmRS≥1) following first-line treatment (NEOS2-T), and (3) return to work/school within three years after diagnosis (NEOS2-W). The NEOS2, NEOS2-T and NEOS2-W scores largely overlap. The numbers are roughly based on data distributions and effect sizes in the three models. Effects of transformed variables, with or without splines and interactions, were non-linear. This effect was captured in the numbers assigned. The attenuating effect of (treatment) delay on leucocyte number was captured in different composite categories. The model included treatment delay as a main effect and part of the interaction with leucocyte count in diagnostic CSF, excluding the highly correlated variable of lumbar puncture timing (Pearson's R = 0.84). As the numbers were based on a model calculation, it was impossible to get the maximum score for CSF leucocyte count (high number of leucocytes and a short treatment delay of <2 weeks, often inherently corresponding to a short delay to the diagnostic lumbar puncture) and the maximum score for treatment delay (>2 months) simultaneously. Total scores therefore add up to a maximum of 10 (NEOS2-T) to 20 (NEOS2-W).

Abbreviations: NEOS2 = the Second anti-NMDAR Encephalitis One-year functional Status score, NEOS2-T = the anti-NMDAR Encephalitis Outcome Score after Treatment, NEOS2-W = the anti-NMDAR Encephalitis Outcome Score for Work or education, ICU = intensive care unit, CSF = cerebrospinal fluid.

**Fig. 3:**
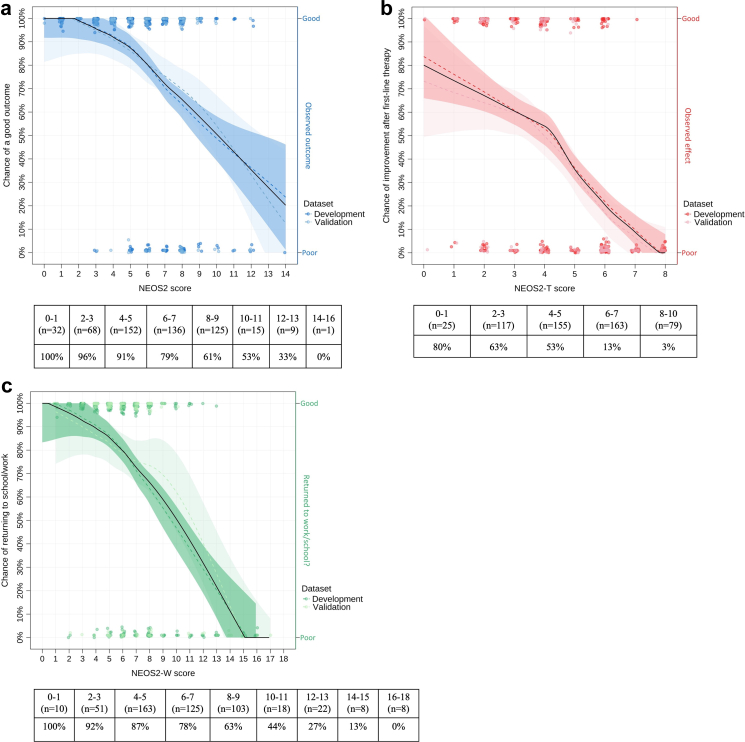
**The NEOS2, NEOS2-T and NEOS2-W models**. Proportion of patients with a certain outcome per NEOS2 score. The dashed lines represent the proportion of patients in the development (dark) and validation (light) cohort obtaining a certain outcome per score, the black line represents the total cohort. The dots are the individual patients with a certain outcome. A: The NEOS2 prediction score, applicable at diagnosis, classifies patients with a very high chance (approaching 100%) of a good functional outcome (mRS≤2) one year after the anti-NMDAR encephalitis diagnosis, versus patients with high risk (80–90%) of a poor outcome. B: The NEOS2-T score, also applicable at diagnosis, classifies patients with a high (80%) chance of improvement after first-line immunotherapy, and patients with an almost definite risk of first-line therapy failure. C: The NEOS2-W score classifies patients with a very high chance (approaching 100%) of returning to work/school within three years after the anti-NMDAR encephalitis diagnosis, and patients very unlikely to resume work or education.

The result is a ‘NEOS2-score’ for predicting middle-long-term functional outcome (Fig. 3A), a ‘NEOS2-T score’ for predicting early improvement following first-line immunotherapy (Fig. 3B) and a ‘NEOS2-W score’ for predicting ability to return-to-work or school within three years (Fig. 3C).

## Discussion

This international cohort study provides several important findings: (1) long-term outcomes of anti-NMDAR encephalitis, as well as who will improve following first-line immunotherapy, can be predicted with high accuracy; (2) the easily applicable NEOS2-score enables early identification, at the time of diagnosis, of patients with an excellent prognosis (almost 100% likelihood of good outcome) and those at high risk (80–90%) of poor outcome; (3) the NEOS2-T score also identifies patients with a high probability (80%) of improving following first-line immunotherapy, as well those who are highly likely to require more aggressive treatment; (4) the predictors of long-term outcomes and improvement following first-line therapy substantially overlap, suggesting that early intensified treatment in patients at risk for first-line failure and poor outcome, may improve long-term functional outcomes; (5) return to work or school can serve as a sensitive indicator of very long-term recovery.

The original NEOS-score predicts functional outcomes one year after diagnosis and treatment of anti-NMDAR encephalitis,^14^ and has been validated as a useful tool.^18^, ^19^, ^20^ However, a limitation is the one-month delay between the initiation of treatment and the earliest time point at which the assessment can be performed. Removing the variable related to treatment-response, as suggested in a published meta-analysis of treatment strategies, demonstrated insufficient predictive accuracy in our cohort (AUC 68%) to support clinical or scientific relevance.^21^ In contrast, the newly developed and validated NEOS2-score is able to predict long-term outcomes at the time of diagnosis, before starting treatment, with the same accuracy as the original NEOS-score.^14^ This represents a substantial and clinically relevant improvement, enhancing its potential implementation in daily practice, and enabling timely identification of patients at risk of a poor outcome.

Interestingly, the prediction for improvement after first-line therapy (NEOS2-T score) included almost the same variables as the NEOS2. Previous studies have shown that patients who do not respond to first-line therapy may benefit from second-line immunotherapy.^1^ Taken together, these findings suggest that patients at risk of poor outcome according to the NEOS2-score may benefit from early intensified treatment, initiated right after the diagnosis. The potential benefits of intensified or second-line immunotherapy must be carefully weighed against the associated risks, including increased susceptibility to infections in vulnerable patients, and individual factors such as age, comorbidities and fertility considerations. The NEOS2-score is also able to identify patients with a good prognosis, who do not require costly and sometimes harmful second-line therapies. We intentionally avoid specifying thresholds for initiating or withholding second-line therapy, as determining the optimal treatment regimen, including the combination of first- and second-line therapies, falls outside the scope of this study. The potential utility of the NEOS2(-T) scores in guiding treatment decisions requires confirmation in future prospective studies. The use of rituximab and cyclophosphamide has been associated with a lower risk of relapses.^1^ The current study was not designed to assess relapse risk systematically, and the relapse risk was not easily predicted by the NEOS2-score (Supplementary Figure S11; accuracy of 60–65%).

The larger dataset and implementation of more complex patterns in the data analysis, such as non-linear and interaction effects, provided more precision and improved accuracy compared to the original NEOS-score analysis.^14^ The NEOS2-scores are slightly different from the original NEOS-score. ICU-requirement, CSF leucocyte count, and treatment delay are predictive variables in both scores. We identified a subset of patients with a marked pleocytosis and a severe onset of clinical symptoms, who were consequently quickly diagnosed and initiated on immunotherapy. Nevertheless, they had a more aggressive disease, poorer response to first-line immunotherapy and outcome at 1 and 3 years than anticipated based on the predictive items, including the time to start immunotherapy. This seemingly paradoxical response is reflected in the interaction items combining pleocytosis and treatment delay. This would suggest that early pleocytosis, in addition to the independent effect of pleocytosis itself, is a marker for the involvement of an additional immune pathway that may convey a larger risk for a poor outcome. The nature of this immune pathway merits further research.

Age and the presence of movement disorders were included in the updated NEOS2-scores, while MRI no longer remained an independent predictive factor. Age has been recognised as an important factor for outcome in several previous studies,^1^^,^^3^^,^^10^ especially in patients older than 30–40 years; the non-linear relationship (Fig. 2) complicated the incorporation in prediction scores. The use of splines, and accordingly, non-linear scoring of age in the prediction model, solved this and partly explains the difference between the original NEOS-score and the NEOS2-scores. As plasticity and vulnerability of the brain, and the ability to participate in active (cognitive) rehabilitation programs and additional (medical) treatments differ by age, it is not surprising that age is an essential factor in the middle- and long-term outcome models (NEOS2 and NEOS2-W). Age did not impact the (early) effectiveness of immunotherapy considerably, probably because this outcome is determined mainly by immunological factors rather than by neurocognitive recovery potential. A lesser dependence of this outcome variable on external factors such as rehabilitation programs and intensity of immunotherapy, might explain the ability to predict it with equal accuracy, using a similar set of predictive variables (excluding age).

We present three scores (NEOS2, NEOS2-T and NEOS2-W) with their slightly unique build-up and weighing, to inform readers about these differences. Depending on the setting, a version can be applied. The overlap of items between the short-term NEOS2-T, the middle-long-term NEOS2 and the long-term NEOS2-W models, and the similar performance of the models in the development and validation cohorts, advocate the credibility of the models. This provides important support for the model's generalisability to new cases. We additionally promoted generalisability by including multicentre cohorts, limiting the performance of the models on paper (AUC; we find a better performance in the monocentric cohorts of the Netherlands, Spain, and Japan) but reflecting real-world data.

Although we present values for the sensitivity and specificity of the NEOS2-scores, these measures of performance depend on a prespecified cut-off of the NEOS2. The use of the ordinal score, allows to overcome some of the limitations in sensitivity or specificity. The high specificity and good sensitivity of the NEOS2-score support its ability to predict poor outcomes accurately; however, some patients predicted to have good outcome may still experience less favourable results. The NEOS2-score provides more nuance, showing a gradual decrease of likelihood of good outcome, ranging from 96 to 100% (score 0–3) to 61% (score 8–9) to <20% (score 14–16). This will provide physicians with relevant information to adequately counsel patients and families. The high sensitivity of the NEOS2-T score allows to identify those likely to have clear benefit of first-line immunotherapy, while the less optimal specificity might lead to escalation in some patients that would have benefited (measured by mRS improvement) using first-line immunotherapy only.

The mRS/PCPC has limitations in measuring improvement after treatment and over time. Not every improvement is captured in the mRS/PCPC. Although patients with an mRS of 3 may remain dependent, seizures or psychotic behaviour may resolve. Similarly, patients in the ICU may recover from autonomic dysregulation without improving on the mRS. This might have led to the relatively large impact of ICU-requirement in the NEOS2-T prediction and potentially to the disqualification of more subtle predictors such as dysautonomia, seizures, or cognitive abilities. The Clinical Assessment Scale in Autoimmune encephalitis (CASE) score may be a more sensitive outcome measure for the acute phase.^22^ These data were not systematically collected in this international cohort but may be worthwhile collecting for future studies. Conversely, especially for the severely affected patients best identified with the NEOS2-T score, subtle clinical improvement may not be as important in guiding treatment strategy; if a patient cannot leave the ICU, although clinically improving, this may still be a reason to intensify treatment early.

**Fig. 4:**
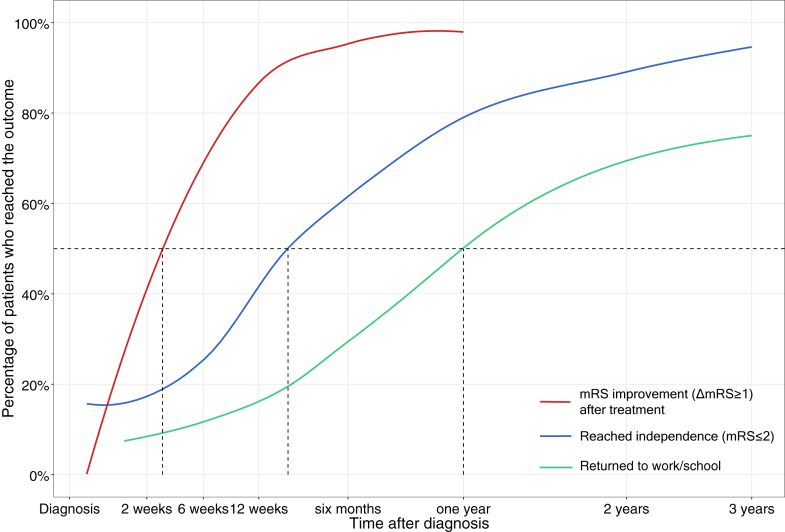
**Outcome measurement as a continuum**. Proportion of patients in our dataset having reached the different incorporated outcomes at the time points specified on the x-axis, derived from the supporting variables on timing of reaching the outcome (Supplementary Data Dictionary). In the first two weeks after first-line treatment, 40% of patients improve one point on the mRS (red line). Measuring outcome in terms of reaching independence (mRS≤2; blue line) may be relevant for the first 6 months to a year after diagnosis, in which 60–80% reach independence. For the longer term, outcome measures that are more sensitive to persistent cognitive and psychosocial sequelae would be more relevant. As a binary substitute, the ability to resume work or school seems a suitable measure (green line). After one year, 50% of patients have resumed work or school; after three years, 75%.

Introducing sensitive long-term outcome measures is imperative.^23^ We and others have demonstrated that cognitive and psychosocial recovery can continue beyond the first one or two years after diagnosis,^7^^,^^8^ when most patients (80–90%) have reached independence, yet only 50–75% have returned to work/school.^24^ This ceiling effect of the standard primary outcome measure (functional independence; mRS 0–2), and consequent skew in outcomes, does not accredit the relevant cognitive and behavioural problems patients still suffer from, and reduces the power of prognostic studies. However, different questions demand different outcome measures. To identify the most severely affected patients who may never reach independence –and might benefit from fast aggressive treatment– a simple, crude measure like the mRS may suffice, while differentiation in the larger part of patients with an expected good outcome with greater detail (and statistical power) by introducing more sensitive outcome measures might be more relevant for the longer term. We introduced ‘ability to return to work/school’ as a readily available, dichotomous measure to assess (long-term) functional outcomes, in addition to initial treatment response and functional independence as short and middle-long term outcomes (Fig. 4). The level of education was not collected in a standardised fashion in this cohort, and whether a considerable proportion of younger children resumed school, but at a lower level, could not be assessed. Previous research has shown that this is unfortunately not uncommon.^5^ We also recognise that the motivation to return to work may decrease nearing retirement, although the influence of age was already significant up from the age of 36.

A limitation of the NEOS2-models is that the models were developed with ‘real world data’. This leads to limitations regarding the availability of data, exact timing of capturing data and variable interpretation of content. At the same time, this can be seen as an advantage, as it makes the models generalisable and relevant in clinical practice. The inclusion of several international cohorts might provide known and unknown confounders, including differences in policy and culture (i.e. reasons for ICU admission, treatment strategies). We decided to fit a mixed-effects model to correct for international differences and proved that, although there were differences between the cohorts at first sight, cohort was not a significant factor after case mix correction. This emphasises the applicability of the developed models across international borders.

In line with the nature of real-world cohort data, the patients were not treated with a pre-specified protocol nor randomised for specific treatments. In clinical practice, treatment is already adjusted for patients who do not improve after first-line therapy.^1^^,^^14^ Patients with characteristics relating to no improvement after first-line therapy, as well as to a poor functional outcome, already received second-line treatment more frequently, albeit often later in the disease course, potentially improving outcomes for these patients. Shifting patients with poor expected outcomes towards better outcomes in this way may have reduced the accuracy and calibration of the NEOS2 and NEOS2-W models. Although this might have affected the reported performance of the models, it would underscore the goal of the NEOS2 and NEOS2-T score: to identify patients prone to fail first-line immunotherapy and at risk for poor outcomes early and before awaiting failure of first-line therapy, reducing the delay of more aggressive treatments.

The use of the NEOS2-score to stratify patients within trials would probably increase the power and reduce needed sample.

Additional biomarkers related directly or indirectly to inflammation such as C-X-C motif chemokine-ligand 10 (CXCL10), CXCL13, C–C motif chemokine ligand 20 (CCL20), CCL22, tumour necrosis factor α (TNF-α), interleukin-6 (IL-6), and IL-17 were not available for analysis. Although these biomarkers may be scientifically informative to understand the pathophysiology better, they are far from applicable to make personalised outcome predictions and treatment strategies.^25^, ^26^, ^27^, ^28^, ^29^ Issues relating to accurate measuring,^29^^,^^30^ and the lack of real-time availability limit their potential use. The items incorporated in the NEOS2-scores are easy to collect and do not harbour these limitations.

### Conclusions

The NEOS2, NEOS2-W and NEOS2-T models can accurately identify patients at risk for a poor outcome, at the middle- and long-term (up to three years), and first-line immunotherapy failure, at the time of anti-NMDAR encephalitis diagnosis. These patients may benefit from early intensified treatment.

## Contributors

Juliette Brenner and Maarten J. Titulaer conceptualised and designed the study. Juliette Brenner collected, curated, and analysed the data, performed the formal analysis, created the visualisations, and drafted the manuscript. Maarten J. Titulaer secured funding, supplied resources, managed the project, supervised the research, validated findings, and provided critical review of the manuscript. Robert van den Berg and Victor Volovici provided input on the methodology and reviewed the manuscript. Sharon Veenbergen and Marco W.J. Schreurs supplied resources, collected and curated data, and reviewed the manuscript. Peter A.E. Sillevis Smitt was responsible for resources, administration, and review. Anna E.M. Bastiaansen, Mar Guasp, Sergio Muiz-Castrillo, prof. Takahiro Iizuka, Marienke A.A.M. de Bruijn, Amaia Muñoz-Lopetegi, Eugenia Martínez-Hernández, Géraldine Picard, prof. Alberto Vogrig, Mathilde Millot, prof. Carsten Finke, Christian Geis, prof. Jan Lewerenz, prof. Nico Melzer, prof. Harald Prüss, Saskia Ruber, Marius Ringelstein, prof. Kevin Rostsy, prof. Kurt-Wolfram Shös, Franziska S. Thaler, Prof. Klaus-Peter Wandinger, Katharina Wurdack, Yvette S. Crijnen, Jeroen Kerstens, Robin W. van Steenhoven, Rinze F. Neuteboom, Juna M. de Vries, Suzanne C. Franken, Mariska M.P. Nagtzaam, prof. Josep Dalmau, prof. Frank Leypoldt, and prof. Jérôme Honnorat, and members of the GENERATE study group, contributed to data acquisition, data curation, and reviewing.

Juliette Brenner and Maarten J. Titulaer verified the data. All authors had full access to the data, and all authors take responsibility for the decision to submit the manuscript for publication.

## Data sharing statement

Data collected for the study, including anonymised individual participant data and a data dictionary defining each field in the set, will be made available with this publication on reasonable request from any qualified investigator, reviewed by the first and senior author of this manuscript and the principal investigators of the respective cohorts. Proposals may be directed to the corresponding author.

## Declaration of interests

J. Brenner and M. J. Titulaer hold a copyright, on behalf of Erasmus MC, for the patient-reported outcome measure PROSE.

J. Brenner is supported by the AEA Seed Grant 2024.

E. Martínez-Hernández is supported by grants from the Instituto de Salud Carolos III (PI20-00820) and Obra Social Fundación la Caixa (CI24-20499). E. Martínez-Hernández has received honoraria for lectures and travel expenses for attending meetings from UCB Pharma, Terumo, Janssen, CSL Behring and Alexion Pharmaceuticals, and is on the Advisory board of Argenx, Alexion and UCB Pharma; none related to this study.

C. Finke is supported by grants from the Deutsche Forschungsgemeinschaft (Heisenberg Program, FI 2309/1-1, FI 2309/2-1; SFB 1315, 327654276; Clinical Research Unit KFO 5023 ‘BecauseY’, 504745852).

C. Geis has received royalties for consulting from Soby and Kyowa, honoraria for lectures and travel expenses for attending meetings from Roche, Argenx, Alexion and AstraZeneka, and is on the Advisory board of Roche, Alexion, and Argenx; none related to this study.

N. Melzer is supported by grants from Euroimmun, Fresenius Medical Care, Diamed, Alexion Pharmaceuticals, and Novartis Pharma. N. Melzer has received honoraria for lectures and travel expenses for attending meetings from Biogen Idec, GlaxoSmith Kline, Teva, Novartis Pharma, Bayer Healthcare, Genzyme, Alexion Pharmaceuticals, Fresenius Medical Care, Diamed, UCB Pharma, AngeliniPharma, BIAL and Sanofi-Aventis, and has received royalties for consulting from UCB Pharma, Alexion Pharmaceuticals and Sanofi-Aventis; none related to this study.

S. Räuber received travel grants from Merck Healthcare Germany GmbH, Alexion Pharmaceuticals, and Bristol Myers Squibb, and served on a scientific advisory board from Merck Healthcare Germany GmbH and received honoraria for lecturing from Roche and Merck Healthcare Germany GmbH. Her research was supported by Novartis Pharma GmbH, Sanofi-Aventis Deutschland GmbH, ‘Stiftung zur Förderung junger Neurowissenschaftler‘, and ‘Else Kröner-Fresenius-Stiftung‘; none related to this study.

M. Ringelstein received speaker honoraria and travel reimbursement from Roche, Alexion, and Horizon/Amgen, and is on the Advisory Board of Roche, Alexion, and Horizon/Amgen; none related to this study.

K. Rostàsy has received consulting fees from Roche (Operetta2study) and Merck; none related to this study.

K.W. Sühs has received honoraria for lectures and travel reimbursements for attending meetings from Bavarian Nordic, Biogen, Bristol-Myers, Merck, Mylan, Novartis, Roche, Viatris and Bristol-Myers Squibb; none related to this study.

F.S. Thaler has received speaker honoraria from Alexion.

R.W. van Steenhoven is supported by ZonMW (VIMP program), Alzheimer Nederland (Interact Grant 2023), an AEA Seed Grant (2023), Erasmus University Medical Center Innovation Grant (2024), a van Leersum Travel Grant from the Royal Academy of Science (2024) and the ItsME foundation.

J.M. de Vries is the local investigator at the Erasmus MC for the Cielo study; a phase III trial by F. Hoffmann-La Roche Ltd/Chugai Pharmaceutical Co. Ltd.

P.A.E. Sillevis Smitt has a patent for the detection of anti-DNER and receives royalties from Euroimmun for diagnostic use of the anti-DNER patent. P.A.E. Sillevis Smitt has an unrestricted grant from Euroimmun. None related to this study.

J. Dalmau is supported by grants from Fundació Clinic per a la Recerca Biomedica, Caixa Research Institute and Instituto de Salud Carlos III (PI23/00858 and PI20/00197). J. Dalmau has royalties for the use of NMDAR as an antibody test. None related to this study.

F. Leypoldt is supported by the German Ministry of Education and Research BMBF for the GENERATE registry (01GM1908A & 01GM2208). F. Leypoldt is supported by E-Rare Joint Transnational research support (ERA-Net, LE3064/2-1), Stiftung Pathobiochemie of the German Society for Laboratory Medicine, European Joint Program for Neurodegenerative Diseases (EJPRD) IGNITEMIND (01ED2506B), ERA-Net MICE-AE (01EW2507B) and HORIZON MSCA 2022 Doctoral Network 101119457 - IgG4-TREAT.

F. Leypoldt discloses speaker honoraria from Grifols, Teva, Argenx, Biogen, Bayer, Roche, Novartis, Fresenius, travel funding from Grifols and serving on advisory boards for Roche, Biogen, Argenx and Alexion; none related to this study.

M.J. Titulaer is supported by grants from Dioraphte (2001 0403), EpilepsieNL (19-08), the Dutch Research Council (ZonMW, PARADE-VIMP, JPND, IgniteMIND, ActMD), E-RARE JTC 2018 (UltraAIE 90030376505), ItsME, the Erasmus Trust foundation, the Erasmus MC foundation, and the Interlaken Leadership award. M.J. Titulaer has royalties from UpToDate inc. and a patent for the detection of KTCD16 antibodies. He is on the scientific advisory boards of Encephalitis International, the Autoimmune Encephalitis Alliance and ItsME. He is on the editorial boards of Neurology: Neuroimmunology & Neuroinflammation and the Dutch Journal for Neurology & Neurosurgery (TNN). He has received funds for serving on scientific advisory boards of AmGen, UCB, Arialys and ArgenX.

A.E.M. Bastiaansen, M. Guasp, M.A.A.M. de Bruijn, S. Muñiz-Castrillo, T. Iizuka, A. Muñoz-Lopetegi, G. Picard, A. Vogrig, M. Millot, J. Lewerenz, H. Prüss, F. Thaler, K.P. Wandinger, K. Wurdack, Y.S. Crijnen, J. Kerstens, S. Veenbergen, M.W.J. Schreurs, *R.* van den Berg, V. Volovici, R.F. Neuteboom, M.M.P. Nagtzaam, S.C. Franken J. Dalmau, and J. Honnorat report no disclosures related to this work.
