# Supplementary material for: Development and validation of the NEOS2 score for prediction of long-term outcomes and improvement after first-line immunotherapy in patients with anti-NMDAR encephalitis: an international cohort study
Source: Lancet Reg Health Eur. 2025 Dec 11;62:101562. doi: 10.1016/j.lanepe.2025.101562 (PMC12757447; doi:10.1016/j.lanepe.2025.101562)
Supplement: Supplementary Material 1 [file mmc1.docx]

**DATA DICTIONARY**

| **Predictive variables** | **Definition** | **Type** | **Missing (%)** | **Distribution** | **Transformation** |
| --- | --- | --- | --- | --- | --- |
| Sex | Biological sex. | Binary | <1 | - | - |
| Age diagnosis | Age at diagnosis in years. | Continuous | 1 | Positive skew | Square root |
| Tumour | Presence of a tumour within 24 months of diagnosis, potentially related to the AE. | Binary | 3 | - | - |
| Altered consciousness | At least two points decrease on the Glasgow Coma scale at diagnosis. | Binary | 1 | - | - |
| Seizures | Focal or generalized seizures in the days before/at diagnosis. | Binary | 1 | - | - |
| Cognitive disturbances | Estimated by the treating physician. | Binary | 1 | - | - |
| Behavioural changes | Observed by the treating physician, patient or their surroundings. | Binary | 1 | - | - |
| Language disorders | Mutism, aphasia. | Binary | 1 | - | - |
| Movement disorders | Catatonia, dystonia, myoclonus, ataxia, ocular movement disorders by the treating physician. | Binary | <1 | - | - |
| Autonomic dysregulation | Sudden changes in heart rate or blood pressure, sweating profusely. | Binary | 2 | - | - |
| Sleep disorders | Hypersomnia, hyposomnia or more restlessly/vivid dreaming. | Binary | 6 | - | - |
| Severity (mRS) at diagnosis | Modified Rankin Score estimated by the treating physician at diagnosis. | Ordinal | 2 |  |  |
| ICU | Requiring ICU admission before or at diagnosis; potential causes may be dysautonomia, seizures, uncontrollable (psychotic) behaviour. | Binary | 1 | - | - |
| MRI abnormalities | Any abnormalities potentially related to the encephalitis (i.e. hippocampal sclerosis, meningeal enhancement) on an MRI acquired < 3 months from symptom onset. | Binary | 8 | - | - |
| EEG abnormalities | Any abnormalities potentially related to the encephalitis (i.e. epileptic activity, extreme delta brushes, slowing, not explained by medication) on an EEG acquired < 3 months from symptom onset. | Binary | 13 | - | - |
| - Abnormal posterior rhythm | Abnormalities potentially related to the encephalitis in the parieto-occipital leads of an EEG acquired < 3 months from symptom onset. | Binary | 15 | - | - |
| CSF leukocyte count | Total number of leukocytes x10^6^/L in diagnostic CSF, acquired < 3 months from symptom onset. | Continuous | 5 | Positive skew | Log2 |
| - Lumbar puncture timing | Weeks from symptom onset to diagnostic lumbar puncture | Continuous | 3 | Positive skew | Log2 |
| CSF antibody titre | Maximum dilution of diagnostic CSF (< 3 months from symptom onset) in which anti-NMDAR antibodies are still detectable (1 in …). | Ordinal | 36 | - | - |
| Diagnostic delay | Weeks from symptom onset to serologically confirmed diagnosis. | Continuous | 2 | Positive skew | Winsorized & Log2 |
| Treatment delay | Weeks from symptom onset to first type of therapy for anti-NMDAR encephalitis. | Continuous | 2 | Positive skew | Log2 |
| **Treatment variables** |  |  |  |  |  |
| Intravenous methylprednisolone | Received intravenous methylprednisolone (IVMP) as a first-line treatment. | Binary | <1 | - | - |
| Intravenous immunoglobulins | Received intravenous immunoglobulins (IVIG) as a first-line treatment. | Binary | <1 | - | - |
| Plasmapheresis | Plasmapheresis as a first-line treatment. | Binary | <1 | - | - |
| First-line treatment | Any combination of the above-mentioned treatments (IVMP, IVIG and/or plasmapheresis) | Binary | <1 | - | - |
| Second-line therapy | Rituximab, Cyclophosphamide | Binary | <1 | - | - |
| **Outcome variables** |  |  |  |  |  |
| Improved after first-line therapy^1^ | One-point improvement on the mRS ≤ 2weeks after first-line treatment. | Binary | 12 | - | - |
| - Time to first improvement^3^ | Number of days after treatment onset to first improvement (one point on the mRS) | Continuous | 25 | Positive skew | Not applicable |
| Functional outcome^1^ | Dichotomised modified Rankin Score, estimated by the physician/researcher one year (± 1 month) after diagnosis. For paediatric patients, Paediatric Cerebral Performance Category score. | Binary (1 for mRS≤2, 0 >2) | 10 | - | - |
| Reached independence^3^ | Reached mRS≤2 during follow-up. | Binary | 8 | - | - |
| - Time to independence^3^ | Months from diagnosis until reaching mRS≤2. | Continuous | 23 | Positive skew | Log2 |
| Return to work/school^2^ | Returned to work/school during follow-up. | Binary | 15 | - | - |
| -Time to return to work^3^ | Months from diagnosis until returning to work/school. | Continuous | 22 | Positive skew | Square root |
| Total follow-up time^3^ | Months from diagnosis or first-line treatment to last follow-up. | Continuous | 2 | Positive skew | Square root |
| 1. Primary outcome measure, 2. Secondary outcome measure, 3. Variable providing context on (timing of) the outcomes. | | | | | |

**SUPPLEMENTARY TABLES**

**Supplementary table 1: Baseline and disease characteristics of the five included cohorts differed.**

|  | **France**, N=324 | **Germany**, N=170 | **Netherlands**, N=132 | **Spain**, N=31 | **Japan**, N=45 | **p-value**^2^ |
| --- | --- | --- | --- | --- | --- | --- |
| **Baseline characteristics** |  |  |  |  |  |  |
| Sex (female), n / N (%) | 260 / 324 (80) | 135 / 169 (80) | 103 / 132 (78) | 24 / 31 (77) | 35 / 45 (78) | >0·99^3^ |
| Year diagnosis, Median (IQR, Min.-Max.) | 2014 (‘11-‘17,  2007-2020) | 2014 (‘12-‘17,  2007-2021) | 2016 (‘13-‘18,  2007-2021) | 2018 (‘17-‘19,  2016-2020) | 2015 (‘11-‘17,  2007-2019) | <0·0001*^4^ |
| Age disease onset, Mean (SD, CI, Min.-Max.) ^1^ | 19 (2, 2-65, 1-80) | 26 (2, 7-67, 3-75) | 28 (3, 3-73, 1-87) | 24 (2, 5-53, 4-56) | 28 (1, 12-55, 12-63) | <0·0001*^5^ |
| Follow-up time (months), Mean (SD, CI, Min.-Max.) ^1^ | 25 (0, 4-97, 0-133) | 39 (1, 12-121, 4-263) | 42 (1, 12-162, 4-366) | 17 (0, 7-32, 4-34) | 31 (1, 4-108, 1-151) | <0·0001*^5^ |
| **Clinical variables** |  |  |  |  |  |  |
| Tumour, n / N (%) | 89 / 310 (29) | 36 / 165 (22) | 37 / 131 (28) | 8 / 31 (26) | 22 / 45 (49) | 0·015*^6^ |
| Altered consciousness at presentation, n / N (%) | 182 / 322 (57) | 100 / 170 (59) | 54 / 132 (41) | 8 / 28 (29) | 39 / 45 (87) | <0·0001*^6^ |
| Seizures, n / N (%) | 266 / 323 (82) | 106 / 167 (63) | 85 / 132 (64) | 16 / 29 (55) | 39 / 45 (87) | <0·0001*^6^ |
| Cognitive disturbances, n / N (%) | 294 / 323 (91) | 148 / 164 (90) | 117 / 132 (89) | 26 / 29 (90) | 43 / 45 (96) | 0·78^6^ |
| Behavioural changes, n / N (%) | 310 / 324 (96) | 140 / 163 (86) | 126 / 132 (95) | 29 / 29 (100) | 44 / 45 (98) | 0·0022*^6^ |
| Language disorders, n / N (%) | 222 / 321 (69) | 86 / 168 (51) | 88 / 132 (67) | 23 / 29 (79) | 38 / 45 (84) | <0·0001*^6^ |
| Movement disorders, n / N (%) | 234 / 323 (72) | 67 / 170 (39) | 74 / 132 (56) | 19 / 29 (66) | 36 / 45 (80) | <0·0001*^6^ |
| Autonomic dysregulation, n / N (%) | 144 / 320 (45) | 63 / 164 (38) | 59 / 131 (45) | 11 / 29 (38) | 31 / 45 (69) | 0·0080*^6^ |
| Sleep disorders, n / N (%) | 123 / 319 (39) | 64 / 143 (45) | 57 / 128 (45) | 28 / 29 (97) | 9 / 44 (20) | <0·0001*^6^ |
| Severity (mRS) at diagnosis, Median (IQR, Min.-Max.) ^1^ | 4 (3-5, 1-5) | 3 (3-4, 1-5) | 3 (3-4, 1-5) | 4 (3-5, 3-5) | 5 (4-5, 3-5) | <0·0001*^4^ |
| Maximum disease severity (mRS), Median (IQR, Min.-Max.) ^1^ | 5 (4-5, 1-6) | 4 (3-5, 1-5) | 4 (3-5, 3-5) | 4 (4-5, 2-5) | 5 (5-5, 3-5) | <0·0001*^4^ |
| ICU admission, n / N (%) | 196 / 320 (61) | 77 / 169 (46) | 63 / 132 (48) | 16 / 31 (52) | 30 / 45 (67) | 0·0020*^6^ |
| **Ancillary tests** |  |  |  |  |  |  |
| MRI abnormalities, n / N (%) | 90 / 299 (30) | 66 / 151 (44) | 30 / 120 (25) | 7 / 30 (23) | 12 / 43 (28) | 0·0090*^6^ |
| EEG abnormalities, n / N (%) | 285 / 285 (100) | 107 / 144 (74) | 95 / 112 (85) | 22 / 28 (79) | 32 / 42 (76) | <0·0001*^6^ |
| - Abnormalities in posterior rhythm, n / N (%) | 184 / 284 (65) | 69 / 144 (48) | 39 / 97 (40) | 13 / 27 (48) | 27 / 42 (64) | <0·0001*^6^ |
| CSF leukocyte count (x10^6^/L), Mean (SD, CI, Min.-Max.)^1^ | 19 (5, 1-244, 1-700) | 21 (5, 1-392, 1-562) | 20 (4, 1-247, 1-848) | 11 (4, 1-121, 1-200) | 34 (4, 2-299, 1-553) | 0·043*^5^ |
| CSF antibody titre (1 in …), Median (IQR, Min.-Max.) ^1^ | - (---) | 32 (10-100, 1-2560) | 32 (13-128, 1-10240) | 48 (32-64, 32-80) | - (---) | 0·42^4^ |
| **Diagnosis & Treatment** |  |  |  |  |  |  |
| Diagnostic delay (weeks), Mean (SD, CI, Min.-Max.)^1^  x.) | 5 (2, 1-26, 0-430) | 3 (3, 1-26, 1-216) | 4 (3, 1-26, 0-835) | 6 (2, 1-22, 1-30) | 9 (3, 1-26, 1-539) | <0·0001*^5^ |
| Treatment delay (weeks), Mean (SD, CI, Min.-Max.)^1^ | 3 (3, 1-47, 0-229) | 3 (4, 1-94, 0-192) | 4 (3, 1-26, 0-68) | 3 (2, 1-14, 1-17) | 2 (3, 0-43, 0-42) | 0·17*^5^ |
| Received first-line immunotherapy, n / N (%) | 312 / 324 (96) | 162 / 170 (95) | 127 / 132 (96) | 30 / 31 (97) | 41 / 45 (91) | 0·52^5^ |
| - Intravenous methylprednisolone (IVMP), n / N (%) | 256 / 324 (79) | 149 / 169 (88) | 121 / 132 (92) | 28 / 31 (90) | 41 / 45 (91) | 0·0023*^5^ |
| - Intravenous immunoglobulins (IVIG), n / N (%) | 293 / 324 (90) | 89 / 168 (53) | 106 / 131 (81) | 26 / 31 (84) | 34 / 45 (76) | <0·0001*^5^ |
| - Plasmapheresis, n / N (%) | 81 / 324 (25) | 100 / 168 (60) | 11 / 131 (8·4) | 7 / 31 (23) | 19 / 45 (42) | <0·0001*^5^ |
| Second-line therapy, n / N (%) | 226 / 324 (70) | 95 / 170 (56) | 59 / 131 (45) | 27 / 31 (87) | 22 / 45 (49) | <0·0001*^5^ |
| (Subjective) effect of first-line therapy, n / N (%) | 156 / 295 (53) | 124 / 153 (81) | 60 / 127 (47) | 11 / 26 (42) | 20 / 41 (49) | <0·0001*^5^ |
| Improvement two weeks after first line therapy, n / N (%) | 78 / 279 (28) | 76 / 143 (53) | 55 / 125 (44) | 13 / 27 (48) | 11 / 41 (27) | <0·0001*^5^ |

| **Long-term outcome** | (n=297) | (n=164) | (n=130) | (n=26) | (n=42) |  |
| --- | --- | --- | --- | --- | --- | --- |
| Outcome (mRS) 12 months after diagnosis, Median (IQR, Min.-Max.) | 1 (0-2, 0-6) | 1 (1-2, 0-5) | 2 (1-2, 0-6) | 1 (0-2, 0-2) | 2 (0-3, 0-5) | <0·0001*^4^ |
| Patients with a good outcome (mRS≤2) after 12 months, n / N (%) | 236 / 288 (82) | 130 / 159 (82) | 99 / 130 (76) | 26 / 26 (100) | 26 / 41 (63) | 0·0021*^6^ |
| Reached independence (mRS≤2) during follow-up, n / N (%) | 251 / 290 (87) | 155 / 159 (97) | 112 / 130 (86) | 26 / 26 (100) | 36 / 42 (86) | 0·0001*^6^ |
| - Time to independence (months), Mean (SD, CI, Min.-Max.)^1^ | 4 (2, 1-19, 1-48) | 3 (3, 1-33, 1-47) | 3 (2, 1-12, 1-23) | 2 (2, 1-7, 1-8) | 7 (3, 2-36, 1-40) | <0·0001*^5^ |
| Ability to go back to work/school, n / N (%) | 204 / 271 (75) | 102 / 129 (79) | 83 / 129 (64) | 23 / 23 (100) | 30 / 42 (71) | 0·0009*^6^ |
| - Time to return to work (months), Mean (SD, CI, Min.-Max.)^1^ | 8 (1, 1-37, 0-76) | 9 (1, 1-63, 0-92) | 9 (0, 1-35, 0-60) | 6 (0, 1-28, 0-29) | - (-, ---) | 0·32^5^ |
| Relapse, n / N (%) | 34 / 285 (12) | 37 / 155 (24) | 22 / 129 (17) | 2 / 26 (7·7) | 6 / 40 (15) | 0·020*^6^ |
| - Time to relapse (months), Mean (SD, CI, Min.-Max.)^1^ | 20 (3, 3-97, 3-112) | 14 (2, 4-74, 3-75) | 30 (3, 4-230, 3-366) | 17 (-, 17-17, 17-17) | 17 (2, 9-29, 9-30) | 0·16^5^ |
| Abbreviations: mRS = modified Rankin Scale, CSF = cerebrospinal fluid, n = number of participants with the feature, N = total number of participants with data, SD = standard deviation, IQR = interquartile range, CI = 95%-confidence interval, Min. = minimum, Max. = maximum | | | | | | |
| ^1^Geometric mean and standard deviation, ^2^*p<0.05; ^3^Pearson's Chi-squared test, ^4^Kruskal-Wallis rank sum test, ^5^one-way ANOVA, ^6^Fisher's exact test | | | | | | |

**Supplementary table 2: The development and validation parts of the original dataset were not significantly different.**

|  | **Development part original data**, N=494 | **Validation part original data**, N=208 | **p-value**^2^ | |
| --- | --- | --- | --- | --- |
| **Baseline characteristics** |  |  |  |  |
| Cohort, n (%) |  |  | >0·99^3^ | |
| - France | 229 (46%) | 101 (47%) |  | |
| - Germany | 120 (24%) | 50 (23%) |  | |
| - the Netherlands | 92 (18%) | 40 (19%) |  | |
| - Spain | 21 (4·2%) | 10 (4·7%) |  | |
| - Japan | 32 (6·4%) | 13 (6·1%) |  | |
| Sex (female), n / N (%) | 397 / 493 (81) | 160 / 208 (77) | 0·28^3^ | |
| Age disease onset, Mean (SD, CI, Min.-Max.)^1^ | 23 (2, 3-69, 1-87) | 23 (2, 3-68, 0-76) | 0·63^5^ | |
| Follow-up time (months), Mean (SD, CI, Min.-Max.)^1^ | 26 (2, 0-126, 0-169) | 27 (2, 0-128, 0-263) | 0·82^5^ | |
| **Clinical variables** |  |  |  |  |
| Tumour, n / N (%) | 138 / 482 (29) | 54 / 200 (27) | 0·67^3^ | |
| Altered consciousness at presentation, n / N (%) | 274 / 491 (56) | 109 / 206 (53) | 0·48^3^ | |
| Seizures, n / N (%) | 361 / 489 (74) | 151 / 207 (73) | 0·81^3^ | |
| Cognitive disturbances, n / N (%) | 438 / 487 (90) | 190 / 206 (92) | 0·34^3^ | |
| Behavioural changes, n / N (%) | 452 / 487 (93) | 197 / 206 (96) | 0·16^3^ | |
| Language disorders, n / N (%) | 312 / 488 (64) | 145 / 207 (70) | 0·068^3^ | |
| Movement disorders, n / N (%) | 301 / 493 (61) | 129 / 206 (63) | 0·70^3^ | |
| Autonomic dysregulation, n / N (%) | 218 / 484 (45) | 90 / 205 (44) | 0·78^3^ | |
| Sleep disorders, n / N (%) | 194 / 469 (41) | 87 / 194 (45) | 0·41^3^ | |
| Severity (mRS) at diagnosis, Median (IQR, Min.-Max.) | 3 (3-5, 1-5) | 3 (3-4, 1-5) | >0·99^4^ | |
| ICU admission, n / N (%) | 274 / 490 (56) | 108 / 207 (52) | 0·36^3^ | |
| **Ancillary tests** |  |  |  |  |
| MRI abnormalities, n / N (%) | 151 / 454 (33) | 54 / 189 (29) | 0·25^3^ | |
| EEG Abnormalities, n / N (%) | 379 / 426 (89) | 162 / 185 (88) | 0·62^3^ | |
| - Abnormalities in posterior rhythm, n / N (%) | 233 / 410 (57) | 99 / 184 (54) | 0·55^3^ | |
| CSF leukocyte count (x106/L), Mean (SD, CI, Min.-Max.)^1^ | 21 (4, 1-267, 0-848) | 18 (5, 1-308, 0-562) | 0·23^5^ | |
| CSF antibody titre (1 in …), Median (IQR, Min.-Max.) | 32 (10-100, 1-2560) | 32 (10-100, 1-10240) | 0·87^4^ | |
| **Diagnosis & Treatment** |  |  |  |  |
| Diagnostic delay (weeks), Mean (SD, CI, Min.-Max.)^1^ | 4 (3, 1-26, 1-26) | 5 (3, 1-26, 1-26) | 0·60^5^ | |
| Treatment delay (weeks), Mean (SD, CI, Min.-Max.)^1^ | 3 (3, 0-46, 0-192) | 3 (3, 0-58, 0-229) | 0·80^5^ | |
| Received first-line immunotherapy, n / N (%) | 476 / 494 (96) | 196 / 208 (94) | 0·20^3^ | |
| - Intravenous methylprednisolone (IVMP), n / N (%) | 417 / 493 (85) | 178 / 208 (86) | 0·74^3^ | |
| - Intravenous immunoglobulins (IVIG), n / N (%) | 385 / 491 (78) | 163 / 208 (78) | 0·99^3^ | |
| - Plasmapheresis, n / N (%) | 157 / 494 (32) | 61 / 208 (29) | 0·43^3^ | |
| Second-line therapy, n / N (%) | 293 / 493 (59) | 136 / 208 (65) | 0·14^3^ | |
| (Subjective) effect of first-line therapy, n / N (%) | 264 / 452 (58) | 107 / 190 (56) | 0·62^3^ | |
| Improvement two weeks after first line therapy, n / N (%) | 163 / 432 (38) | 70 / 183 (38) | 0·90^3^ | |
| **Long-term outcome** |  |  |  |  |
| Outcome (mRS) twelve months after diagnosis, Median (IQR, Min.-Max.) | 1 (0-2, 0-5) | 1 (0-2, 0-6) | 0·40^4^ | |
| Patients with a good outcome (mRS≤2) after 12 months, n / N (%) | 361 / 451 (80) | 156 / 193 (81) | 0·82^3^ | |
| Reached independence (mRS≤2) during follow-up, n / N (%) | 408 / 453 (90) | 172 / 194 (89) | 0·59^3^ | |
| - Time to independence (months), Mean (SD, CI, Min.-Max.)^1^ | 4 (3, 1-24, 1-48) | 3 (2, 1-18, 1-34) | 0·18^5^ | |
| Ability to go back to work/school, n / N (%) | 308 / 416 (74) | 134 / 178 (75) | 0·68^3^ | |
| - Time to return to work (months), Mean (SD, CI, Min.-Max.)^1^ | 9 (1, 0-44, 0-76) | 7 (1, 0-36, 0-92) | 0·63^5^ | |
| Relapse, n / N (%) | 74 / 445 (17) | 27 / 190 (14) | 0·45^3^ | |
| - Time to relapse (months), Mean (SD, CI, Min.-Max.)^1^ | 20 (3, 3-137, 3-366) | 18 (2, 3-71, 3-76) | 0·63^5^ | |
| Abbreviations: mRS = modified Rankin Scale, CSF = cerebrospinal fluid, n = number of participants with the feature, N = total number of participants with data, SD = standard deviation, IQR = interquartile range, CI = 95%-confidence interval, Min. = minimum, Max. = maximum | | | | |
| ^1^Geometric mean and standard deviation | | | |  |
| ^2^*p<0.05; ^3^Pearson's Chi-squared test, ^4^Wilcoxon rank sum test, ^5^Welch Two Sample t-test | | | |  |

**Supplementary table 3: The development and validation parts of the imputed datasets were not significantly different.**

|  | **Imputed dataset 1** | | | | | **Imputed dataset 2** | | | | **Imputed dataset 3** | | | | **Imputed dataset 4** | | | | **Imputed dataset 5** | | | | | | |
| --- | --- | --- | --- | --- | --- | --- | --- | --- | --- | --- | --- | --- | --- | --- | --- | --- | --- | --- | --- | --- | --- | --- | --- | --- |
|  | **Training**, N=493 | | **Validation**, N=209 | **p-value**^2^ | **Training**, N=493 | | **Validation**, N=209 | **p-value**^2^ | **Training**, N=493 | | **Validation**, N=209 | **p-value**^2^ | **Training**, N=493 | | **Validation**, N=209 | **p-value**^2^ | **Training**, N=493 | | **Validation**, N=209 | **p-value**^2^ | | | |  |
| **Baseline characteristics** | | | | | | | | | | | | | | | | | | | | |  |  |  |  |
| Cohort, n (%) |  | |  | >0·99^3^ |  | |  | >0·99^3^ |  | |  | >0·99^3^ |  | |  | >0·99^3^ |  | |  | >0·99^3^ | | | |  |
| - France | 227 (46%) | | 97 (46%) |  | 226 (46%) | | 98 (47%) |  | 227 (46%) | | 97 (46%) |  | 226 (46%) | | 98 (47%) |  | 228 (46%) | | 96 (46%) |  | | | |  |
| - Germany | 119 (24%) | | 51 (24%) |  | 119 (24%) | | 51 (24%) |  | 120 (24%) | | 50 (24%) |  | 119 (24%) | | 51 (24%) |  | 119 (24%) | | 51 (24%) |  | | | |  |
| - the Netherlands | 92 (19%) | | 40 (19%) |  | 93 (19%) | | 39 (19%) |  | 93 (19%) | | 39 (19%) |  | 94 (19%) | | 38 (18%) |  | 93 (19%) | | 39 (19%) |  | | | |  |
| - Spain | 23 (4·7%) | | 8 (3·8%) |  | 23 (4·7%) | | 8 (3·8%) |  | 22 (4·5%) | | 9 (4·3%) |  | 22 (4·5%) | | 9 (4·3%) |  | 22 (4·5%) | | 9 (4·3%) |  | | | |  |
| - Japan | 32 (6·5%) | | 13 (6·2%) |  | 32 (6·5%) | | 13 (6·2%) |  | 31 (6·3%) | | 14 (6·7%) |  | 32 (6·5%) | | 13 (6·2%) |  | 31 (6·3%) | | 14 (6·7%) |  | | | |  |
| Sex (female), n (%) | 392 (80) | | 166 (79) | 0·98^3^ | 386 (78) | | 172 (82) | 0·23^3^ | 386 (78) | | 172 (82) | 0·23^3^ | 392 (80) | | 166 (79) | 0·98^3^ | 391 (79) | | 167 (80) | 0·88^3^ | | | |  |
| Age onset, Mean (SD, CI, Min.-Max.)^1^ | 23 (2, 3-70, 1-87) | | 23 (2, 2-68, 1-76) | 0·94^5^ | 23 (2, 2-69, 1-87) | | 24 (2, 3-69, 1-73) | 0·71^5^ | 23 (2, 3-70, 1-87) | | 23 (2, 3-67, 1-76) | 0·83^5^ | 23 (2, 2-69, 1-87) | | 24 (2, 5-69, 2-76) | 0·32^5^ | 23 (2, 3-69, 1-87) | | 24 (2, 2-69, 1-73) | 0·65^5^ | | | |  |
| **Clinical variables** | | | | | | | | | | | | | | | | | | | | | | | | |
| Tumour, n (%) | 139 (28) | | 58 (28) | 0·90^3^ | 139 (28) | | 60 (29) | 0·89^3^ | 129 (26) | | 69 (32) | 0·080^3^ | 136 (28) | | 64 (31) | 0·42^3^ | 137 (28) | | 58 (28) | 0·99^3^ | | | |  |
| Altered consciousness, n (%) | 273 (55) | | 113 (54) | 0·75^3^ | 267 (54) | | 118 (56) | 0·58^3^ | 262 (53) | | 124 (59) | 0·13^3^ | 262 (53) | | 123 (59) | 0·16^3^ | 271 (55) | | 115 (55) | 0·99^3^ | | | |  |
| Seizures, n (%) | 360 (73) | | 156 (75) | 0·66^3^ | 369 (75) | | 149 (71) | 0·33^3^ | 359 (73) | | 157 (74) | 0·53^3^ | 361 (73) | | 156 (75) | 0·70^3^ | 371 (74) | | 154 (72) | 0·66^3^ | | | |  |
| Cognitive disturbances, n (%) | 444 (90) | | 187 (89) | 0·81^3^ | 444 (90) | | 189 (90) | 0·88^3^ | 439 (89) | | 193 (92) | 0·18^3^ | 445 (90) | | 189 (90) | 0·95^3^ | 446 (90) | | 188 (90) | 0·83^3^ | | | |  |
| Behavioural changes, n (%) | 460 (93) | | 194 (93) | 0·82^3^ | 461 (94) | | 194 (93) | 0·74^3^ | 461 (94) | | 193 (92) | 0·58^3^ | 463 (94) | | 191 (91) | 0·23^3^ | 460 (93) | | 192 (92) | 0·50^3^ | | | |  |
| Language disorders, n (%) | 324 (66) | | 139 (67) | 0·84^3^ | 320 (64) | | 144 (69) | 0·90^3^ | 315 (64) | | 142 (68) | 0·30^3^ | 320 (65) | | 140 (67) | 0·60^3^ | 322 (65) | | 137 (66) | 0·95^3^ | | | |  |
| Movement disorders, n (%) | 301 (61) | | 129 (62) | 0·87^3^ | 302 (61) | | 129 (62) | 0·91^3^ | 301 (61) | | 129 (62) | 0·87^3^ | 301 (61) | | 130 (62) | 0·91^3^ | 301 (61) | | 129 (62) | 0·87^3^ | | | |  |
| Autonomic dysregulation, n (%) | 222 (45) | | 91 (44) | 0·72^3^ | 219 (44) | | 92 (44) | 0·92^3^ | 220 (45) | | 94 (45) | 0·93^3^ | 221 (45) | | 88 (42) | 0·51^3^ | 225 (46) | | 87 (42) | 0·33^3^ | | | |  |
| Sleep disorders, n (%) | 208 (42) | | 87 (42) | 0·89^3^ | 200 (40) | | 86 (41) | 0·89^3^ | 209 (42) | | 85 (41) | 0·67^3^ | 207 (42) | | 85 (41) | 0·75^3^ | 208 (42) | | 90 (43) | 0·83^3^ | | | |  |
| Severity (mRS) at diagnosis, Median (IQR, Min.-Max.) | 3 (3-4, 1-5) | | 3 (3-5, 1-5) | 0·62^4^ | 3 (3-5, 1-5) | | 4 (3-5, 1-5) | 0·14^4^ | 3 (3-4, 1-5) | | 3 (3-5, 1-5) | 0·75^4^ | 3 (3-5, 1-5) | | 3 (3-5, 1-5) | 0·34^4^ | 3 (3-5, 1-5) | | 3 (3-5, 1-5) | 0·51^4^ | | | |  |
| ICU admission, n (%) | 270 (55) | | 109 (52) | 0·53^3^ | 269 (55) | | 108 (52) | 0·48^3^ | 273 (55) | | 105 (50) | 0·21^3^ | 267 (54) | | 111 (53) | 0·80^3^ | 274 (56) | | 109 (52) | 0·40^3^ | | | |  |
| **Ancillary tests** | | | | | | | | | | | | | | | | | | | | |  |  |  |  |
| MRI abnormalities, n (%) | 160 (32) | | 71 (34) | 0·70^3^ | 151 (31) | | 64 (31) | >0·99^3^ | 166 (34) | | 62 (30) | 0·30^3^ | 167 (34) | | 73 (35) | 0·79^3^ | 161 (33) | | 68 (33) | 0·97^3^ | | | |  |
| EEG Abnormalities, n (%) | 433 (88) | | 183 (88) | 0·92^3^ | 428 (87) | | 181 (87) | 0·94^3^ | 429 (87) | | 183 (88) | 0·84^3^ | 436 (88) | | 179 (86) | 0·30^3^ | 439 (89) | | 179 (86) | 0·20^3^ | | | |  |
| - In posterior rhythm, n (%) | 274 (56) | | 114 (55) | 0·80^3^ | 284 (58) | | 125 (60) | 0·59^3^ | 286 (58) | | 120 (57) | 0·88^3^ | 286 (58) | | 111 (53) | 0·23^3^ | 280 (57) | | 120 (57) | 0·88^3^ | | | |  |
| CSF leukocytes, Mean (SD, CI, Min.-Max.)^1^ | 21 (4, 18-24, 0-848) | | 18 (5, 14-22, 0-300) | 0·26^5^ | 20 (5, 18-23, 0-848) | | 18 (4, 14-22, 0-562) | 0·33^5^ | 20 (5, 18-23, 0-700) | | 20 (5, 16-25, 0-848) | 0·93^5^ | 20 (4, 18-23, 0-848) | | 20 (5, 16-24, 0-562) | 0·78^5^ | 20 (5, 18-23, 0-700) | | 18 (5, 15-22, 0-848) | 0·25^5^ | | | |  |
| **Diagnosis & Treatment** | | | | | | | | | | | | | | | | | | | | | |  |  |  |
| Diagnostic delay, Mean (SD, CI, Min.-Max.)^1^ | 4 (3, 1-26, 1-26) | | 4 (3, 1-26, 1-26) | 0·94^5^ | 4 (3, 1-26, 1-26) | | 5 (3, 1-26, 1-26) | 0·52^5^ | 4 (3, 1-26, 1-26) | | 4 (3, 1-26, 1-26) | 0·92^5^ | 4 (3, 1-26, 1-26) | | 5 (3, 1-26, 1-26) | 0·26^5^ | 4 (3, 1-26, 1-26) | | 4 (3, 1-26, 1-26) | 0·93^5^ | | | |  |
| Treatment delay, Mean (SD, CI, Min.-Max.)^1^ | 3 (3, 0-65, 0-229) | | 3 (3, 0-52, 0-192) | 0·62^5^ | 3 (3, 0-68, 0-229) | | 3 (4, 0-102, 0-166) | 0·45^5^ | 3 (3, 0-65, 0-192) | | 3 (3, 1-44, 0-229) | 0·67^5^ | 3 (3, 0-68, 0-229) | | 3 (3, 0-65, 0-192) | 0·78^5^ | 3 (3, 0-44, 0-192) | | 4 (4, 0-68, 0-229) | 0·14^5^ | | | |  |
| Improvement after first-line therapy, n (%) | 194 (39) | | 82 (39) | 0·98^3^ | 197 (40) | | 79 (38) | 0·59^3^ | 198 (40) | | 79 (38) | 0·56^3^ | 193 (39) | | 81 (39) | 0·92^3^ | 189 (38) | | 79 (37) | 0·89^3^ | | | |  |
| **Long-term outcome** | | | | | | | | | | | | | | | | | | | | |  |  |  |  |
| Good outcome at 12 months, n (%) | | 398 (81) | 169 (81) | 0·97^3^ | 401 (81) | | 163 (78) | 0·31^3^ | 389 (79) | | 171 (82) | 0·38^3^ | 396 (80) | | 170 (81) | 0·76^3^ | 402 (82) | | 164 (77) | 0·35^3^ | | | |  |
| Abbreviations: mRS = modified Rankin Scale, CSF = cerebrospinal fluid, n = number of participants with the feature, N = total number of participants with data, SD = standard deviation, IQR = interquartile range, CI = 95%-confidence interval, Min. = minimum, Max. = maximum | | | | | | | | | | | | | | | | | | | | | | |  |  |
| ^1^Geometric mean and standard deviation, ^2^*p<0.05; ^3^Pearson's Chi-squared test, ^4^Wilcoxon rank sum test, ^5^Welch Two Sample t-test | | | | | | | | | | | | | | | | | | | | |  |  |  |  |

**Supplementary table 4: Univariable analysis of acute predictors for functional (mRS) outcome one year after diagnosis.**

|  | **Patients with a poor outcome (mRS>2)**, N=131 | **Patients with a good outcome (mRS≤2)**, N=522 | **p-value**^2^ |
| --- | --- | --- | --- |
| **Baseline characteristics** |  |  |  |
| Sex (female), n / N (%) | 100 / 127 (79) | 412 / 516 (80) | 0·78^3^ |
| Age disease onset, Mean (SD, CI, Min.-Max.)^1^ | 27 (3, 3-73, 1-87) | 22 (2, 3-63, 1-75) | 0·0020**^5^ |
| **Clinical variables** |  |  |  |
| Tumour, n / N (%) | 45 / 124 (36) | 133 / 506 (26) | 0·027**^3^ |
| Altered consciousness at presentation, n / N (%) | 97 / 127 (76) | 259 / 514 (50) | <0·0001**^3^ |
| Seizures, n / N (%) | 102 / 127 (80) | 367 / 515 (71) | 0·039**^3^ |
| Cognitive disturbances, n / N (%) | 114 / 125 (91) | 467 / 513 (91) | 0·95^3^ |
| Behavioural changes, n / N (%) | 117 / 124 (94) | 482 / 514 (94) | 0·81^3^ |
| Language disorders, n / N (%) | 87 / 126 (69) | 338 / 514 (66) | 0·48^3^ |
| Movement disorders, n / N (%) | 100 / 127 (79) | 298 / 516 (58) | <0·0001**^3^ |
| Autonomic dysregulation, n / N (%) | 82 / 124 (66) | 209 / 516 (41) | <0·0001**^3^ |
| Sleep disorders, n / N (%) | 47 / 120 (39) | 214 / 490 (44) | 0·37^3^ |
| Severity (mRS) at diagnosis, Median (IQR, Min.-Max.) | 5 (3-5, 2-5) | 3 (3-4, 1-5) | <0·0001**^4^ |
| ICU admission, n / N (%) | 98 / 127 (77) | 250 / 513 (49) | <0·0001**^3^ |
| **Ancillary tests** |  |  |  |
| MRI abnormalities, n / N (%) | 43 / 114 (38) | 143 / 478 (30) | 0·11*^3^ |
| EEG abnormalities, n / N (%) | 104 / 111 (94) | 401 / 458 (88) | 0·066*^3^ |
| - Abnormalities in posterior rhythm, n / N (%) | 73 / 108 (68) | 236 / 444 (53) | 0·0070**^3^ |
| CSF leukocyte count (x10^6^/L), Mean (SD, CI, Min.-Max.)^1^ | 41 (4, 2-287, 1-700) | 17 (5, 1-295, 1-848) | <0·0001**^5^ |
| CSF antibody titre (1 in …), Median (IQR, Min.-Max.) | 100 (16-128, 2-10240) | 32 (10-100, 1-2560) | 0·0070**^4^ |
| **Diagnosis & Treatment** |  |  |  |
| Diagnostic delay (weeks), Mean (SD, CI, Min.-Max.)^1^ | 5 (3, 1-26, 1-26) | 4 (3, 1-26, 1-26) | 0·016**^5^ |
| Treatment delay (weeks), Mean (SD, CI, Min.-Max.)^1^ | 3 (4, 0-69, 0-156) | 3 (3, 0-35, 0-192) | 0·054*^5^ |
| Delay tumour resection (days), Means (SD, CI; N) | 9 (67, -53-15; 24) | 46 (133, -22-375; 89) | 0·063 |
| Abbreviations: mRS = modified Rankin Scale, CSF = cerebrospinal fluid, n = number of participants with the feature, N = total number of participants with data, SD = standard deviation, IQR = interquartile range, CI = 95%-confidence interval, Min. = minimum, Max. = maximum | | | |
| ^1^Geometric mean and standard deviation | | | |
| ^2^*p<0.15, **p<0.05; ^3^Pearson's Chi-squared test, ^4^Wilcoxon rank sum test. ^5^Two Sample t-test | | | |

**Supplementary table 5: Univariable analysis of predictors for early recovery, two weeks after first-line treatment.**

|  | **No improvement two weeks after treatment**, N=382 | **Improvement two weeks after first treatment**, N=233 | **p-value**^2^ | | |
| --- | --- | --- | --- | --- | --- |
| **Baseline characteristics** |  |  |  |  |  |
| Sex (female), n / N (%) | 302 / 381 (79) | 177 / 233 (76) | 0·34^3^ | |  |
| Age disease onset, Mean (SD, CI, Min.-Max.)^1^ | 23 (2, 3-68, 1-80) | 24 (2, 2-67, 1-75) | 0·31^5^ | |  |
| **Clinical variables** |  |  |  |  |  |
| Tumour, n / N (%) | 123 / 375 (33) | 50 / 229 (22) | 0·0045**^3^ | |  |
| Altered consciousness at presentation, n / N (%) | 254 / 381 (67) | 94 / 232 (41) | <0·0001**^3^ | |  |
| Seizures, n / N (%) | 300 / 381 (79) | 156 / 232 (67) | 0·0021**^3^ | |  |
| Cognitive disturbances, n / N (%) | 343 / 380 (90) | 216 / 230 (94) | 0·11*^3^ | |  |
| Behavioural changes, n / N (%) | 367 / 381 (96) | 213 / 232 (92) | 0·053*^3^ | |  |
| Language disorders, n / N (%) | 259 / 381 (68) | 152 / 232 (66) | 0·53^3^ | |  |
| Movement disorders, n / N (%) | 286 / 381 (75) | 102 / 233 (44) | <0·0001**^3^ | |  |
| Autonomic dysregulation, n / N (%) | 218 / 378 (58) | 71 / 231 (31) | <0·0001**^3^ | |  |
| Sleep disorders, n / N (%) | 161 / 368 (44) | 93 / 214 (43) | 0·95^3^ | |  |
| Severity (mRS) at diagnosis, Median (IQR, Min.-Max.) | 4 (3-5, 1-5) | 3 (3-4, 1-5) | <0·0001**^4^ | |  |
| ICU admission, n / N (%) | 272 / 381 (71) | 74 / 232 (32) | <0·0001**^3^ | |  |
| **Ancillary tests** |  |  |  |  |  |
| MRI abnormalities, n / N (%) | 112 / 355 (32) | 67 / 216 (31) | 0·90^3^ | |  |
| EEG abnormalities, n / N (%) | 322 / 344 (94) | 168 / 207 (81) | <0·0001**^3^ | |  |
| - Abnormalities in posterior rhythm, n / N (%) | 211 / 331 (64) | 89 / 202 (44) | <0·0001**^3^ | |  |
| CSF leukocyte count (x10^6^/L), Mean (SD, CI, Min.-Max.)^1^ | 29 (4, 1-323, 1-848) | 12 (4, 1-241, 1-553) | <0·0001**^5^ | |  |
| CSF antibody titre (1 in …), Median (IQR, Min.-Max.) | 64 (16-128, 1-10240) | 18 (10-50, 1-2560) | <0·0001**^4^ | |  |
| **Diagnosis & Treatment** |  |  |  |  |  |
| Diagnostic delay (weeks), Mean (SD, CI, Min.-Max.)^1^ | 4 (3, 1-26, 1-26) | 5 (3, 1-26, 1-26) | 0·45^5^ | |  |
| Treatment delay (weeks), Mean (SD, CI, Min.-Max.)^1^ | 3 (3, 0-43, 0-166) | 4 (3, 1-68, 0-192) | 0·061*^5^ | |  |
| Abbreviations: mRS = modified Rankin Scale, CSF = cerebrospinal fluid, n = number of participants with the feature, N = total number of participants with data, SD = standard deviation, IQR = interquartile range, CI = 95%-confidence interval, Min. = minimum, Max. = maximum | | | |  |  |
| ^1^Geometric mean and standard deviation | | | |  |  |
| ^2^*p<0.15, **p<0.05; ^3^Pearson's Chi-squared test, ^4^Wilcoxon rank sum test, ^5^Two Sample t-test | | | |  |  |

**Supplementary table 6: Univariable analysis of acute predictors for returning to work or school within three years after diagnosis.**

|  | **Patients not returning to work/school**, N=164 | **Patients returning to work/school**, N=447 | **p-value**^2^ |
| --- | --- | --- | --- |
| **Baseline characteristics** |  |  |  |
| Sex (female), n / N (%) | 117 / 163 (72) | 368 / 447 (82) | 0·0041**^3^ |
| Age disease onset, Mean (SD, CI, Min.-Max.)^1^ | 32 (3, 3-75, 1-87) | 20 (2, 3-48, 1-68) | <0·0001**^5^ |
| **Clinical variables** |  |  |  |
| Tumour, n / N (%) | 54 / 153 (35) | 116 / 441 (26) | 0·034**^3^ |
| Altered consciousness at presentation, n / N (%) | 99 / 164 (60) | 240 / 444 (54) | 0·16^3^ |
| Seizures, n / N (%) | 118 / 162 (73) | 336 / 445 (76) | 0·50^3^ |
| Cognitive disturbances, n / N (%) | 147 / 162 (91) | 411 / 443 (93) | 0·41^3^ |
| Behavioural changes, n / N (%) | 153 / 163 (94) | 423 / 444 (95) | 0·49^3^ |
| Language disorders, n / N (%) | 99 / 163 (61) | 312 / 445 (70) | 0·029**^3^ |
| Movement disorders, n / N (%) | 105 / 164 (64) | 284 / 446 (64) | 0·94^3^ |
| Autonomic dysregulation, n / N (%) | 87 / 161 (54) | 192 / 441 (44) | 0·022**^3^ |
| Sleep disorders, n / N (%) | 57 / 157 (36) | 199 / 423 (47) | 0·021**^3^ |
| Severity (mRS) at diagnosis, Median (IQR, Min.-Max.) | 4 (3-5, 1-5) | 3 (3-5, 1-5) | 0·31^4^ |
| ICU admission, n / N (%) | 102 / 163 (63) | 239 / 445 (54) | 0·051*^3^ |
| **Ancillary tests** |  |  |  |
| MRI abnormalities, n / N (%) | 54 / 144 (38) | 117 / 416 (28) | 0·035**^3^ |
| EEG abnormalities, n / N (%) | 119 / 136 (88) | 358 / 396 (90) | 0·34^3^ |
| - Abnormalities in posterior rhythm, n / N (%) | 71 / 131 (54) | 220 / 384 (57) | 0·54^3^ |
| CSF leukocyte count (x10^6^/L), Mean (SD, CI, Min.-Max.)^1^ | 25 (5, 1-254, 1-700) | 19 (4, 1-269, 1-848) | 0·10*^5^ |
| CSF antibody titre (1 in …), Median (IQR, Min.-Max.) | 32 (8-128, 1-10 240) | 32 (10-100, 1-2 560) | 0·69^4^ |
| **Diagnosis & Treatment** |  |  |  |
| Diagnostic delay (weeks), Mean (SD, CI, Min.-Max.)^1^ | 5 (3, 1-26, 1-26) | 4 (3, 1-26, 1-26) | 0·027**^5^ |
| Treatment delay (weeks), Mean (SD, CI, Min.-Max.)^1^ | 4 (4, 0-70, 0-166) | 3 (3, 0-43, 0-229) | 0·022**^5^ |
| Abbreviations: mRS = modified Rankin Scale, CSF = cerebrospinal fluid, n = number of participants with the feature, N = total number of participants with data, SD = standard deviation, IQR = interquartile range, CI = 95%-confidence interval, Min. = minimum, Max. = maximum | | | |
| ^1^Geometric mean and standard deviation | | | |
| ^2^*p<0.15, **p<0.05; ^3^Pearson's Chi-squared test, ^4^Wilcoxon rank sum test. ^5^Two Sample t-test | | | |

**SUPPLEMENTARY FIGURES**

**Supplementary figure 1: Correlation matrix of the independent variables.**

**
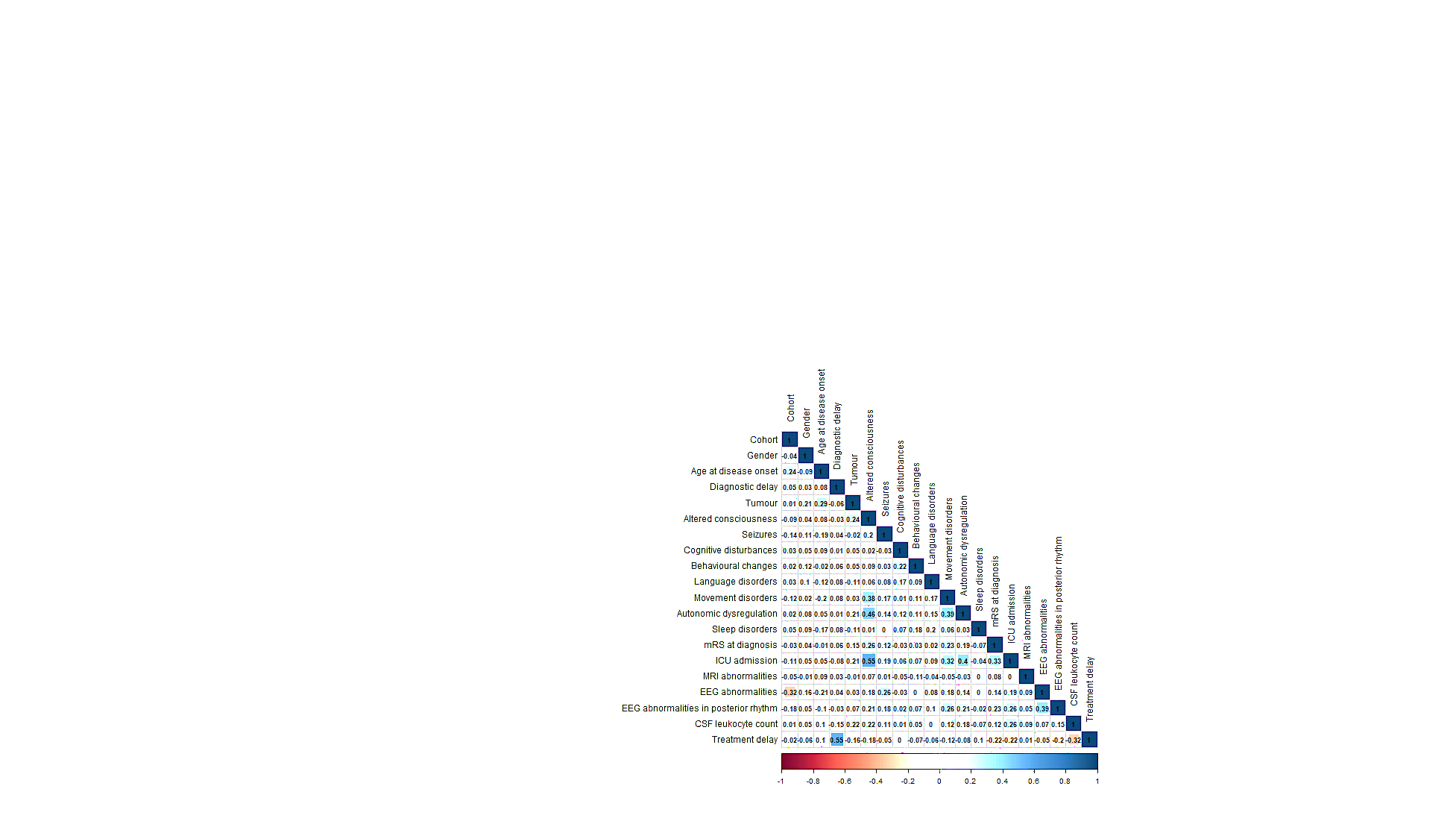
**

**Supplementary figure 2: Interaction effect of CSF leukocyte count and treatment delay in predicting time to independence and time to return to work or school.**


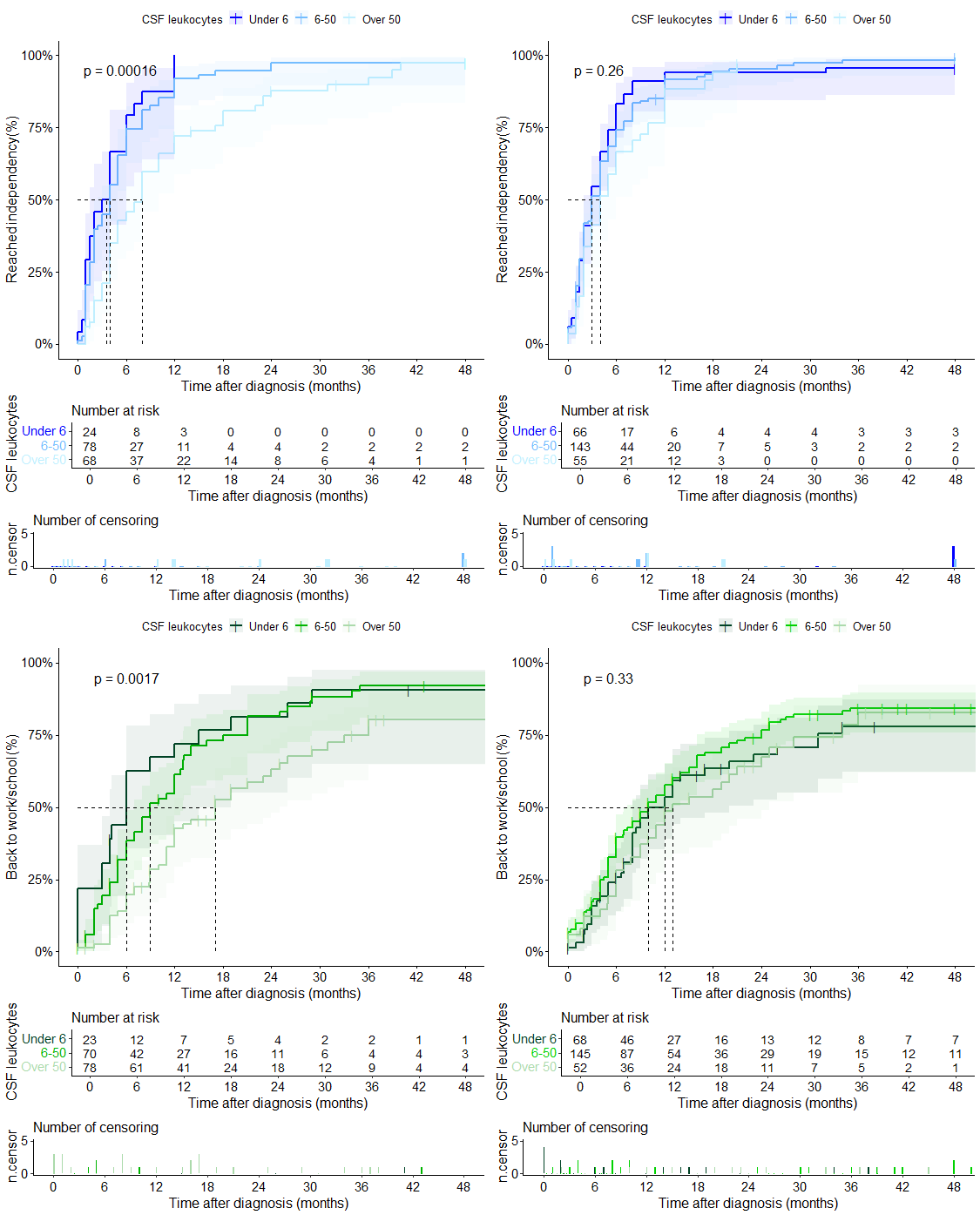


a

b

c

d

Time to independence (A) and time to return to work/school (C) of patients (diagnosed and) treated within two weeks from symptom onset are influenced by CSF leukocyte count. The influence of CSF leukocyte count on recovery rate is more limited in patients diagnosed and treated over two weeks after symptom onset (B & D).

**Supplementary figure 3: ROC curves of the NEOS2 model fitted to the validation parts of the five imputed datasets, displayed per cohort.**

**
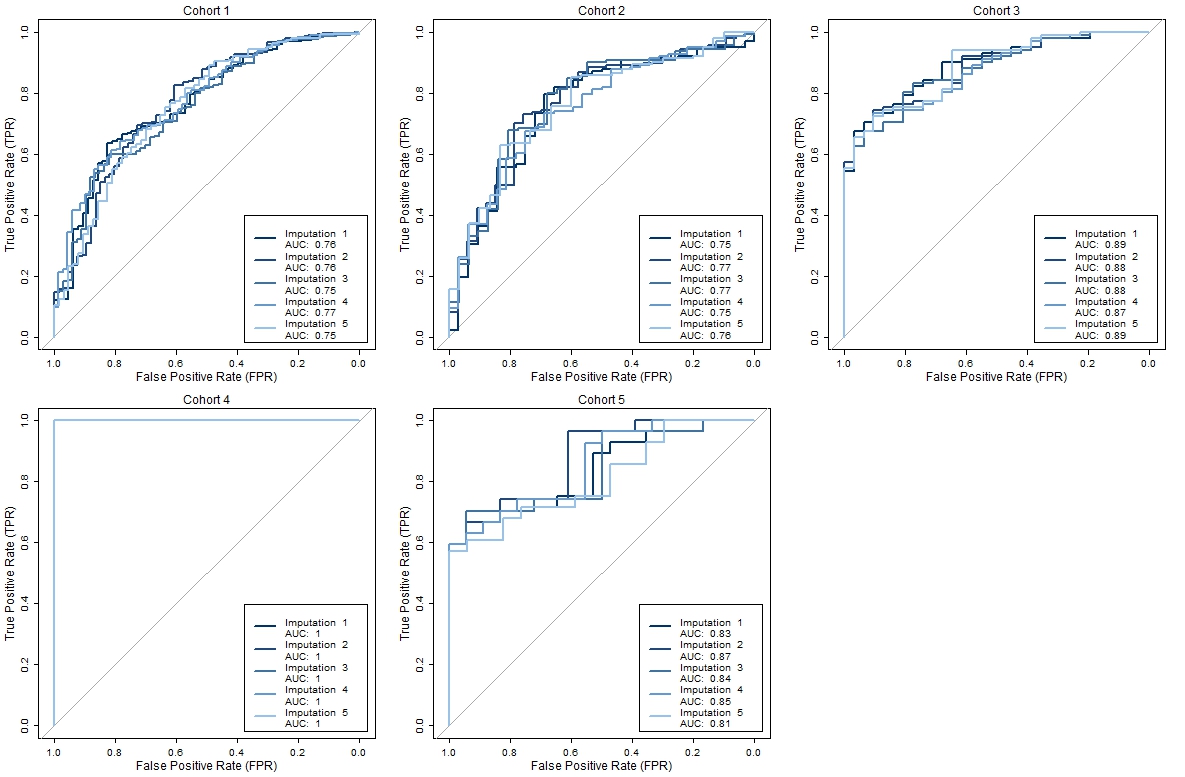
**

Receiver Operator Characteristics of the multivariable model for predicting functional outcome (independence) one year after diagnosis of anti-NMDAR encephalitis (NEOS2 model) fitted to the validation parts of the five imputed datasets, displayed per cohort. Fitting the models to the different imputed datasets yielded similar results across imputations. The model performed well in all five cohorts. Cohort 1=France (N=324), Cohort 2=Germany (N=170), Cohort 3=The Netherlands (N=132), Cohort 4=Spain (N=31), Cohort 5=Japan (N=45).

**Supplementary figure 4: Calibration plot of the multivariable model for functional outcome one year after diagnosis of anti-NMDAR encephalitis.**

**
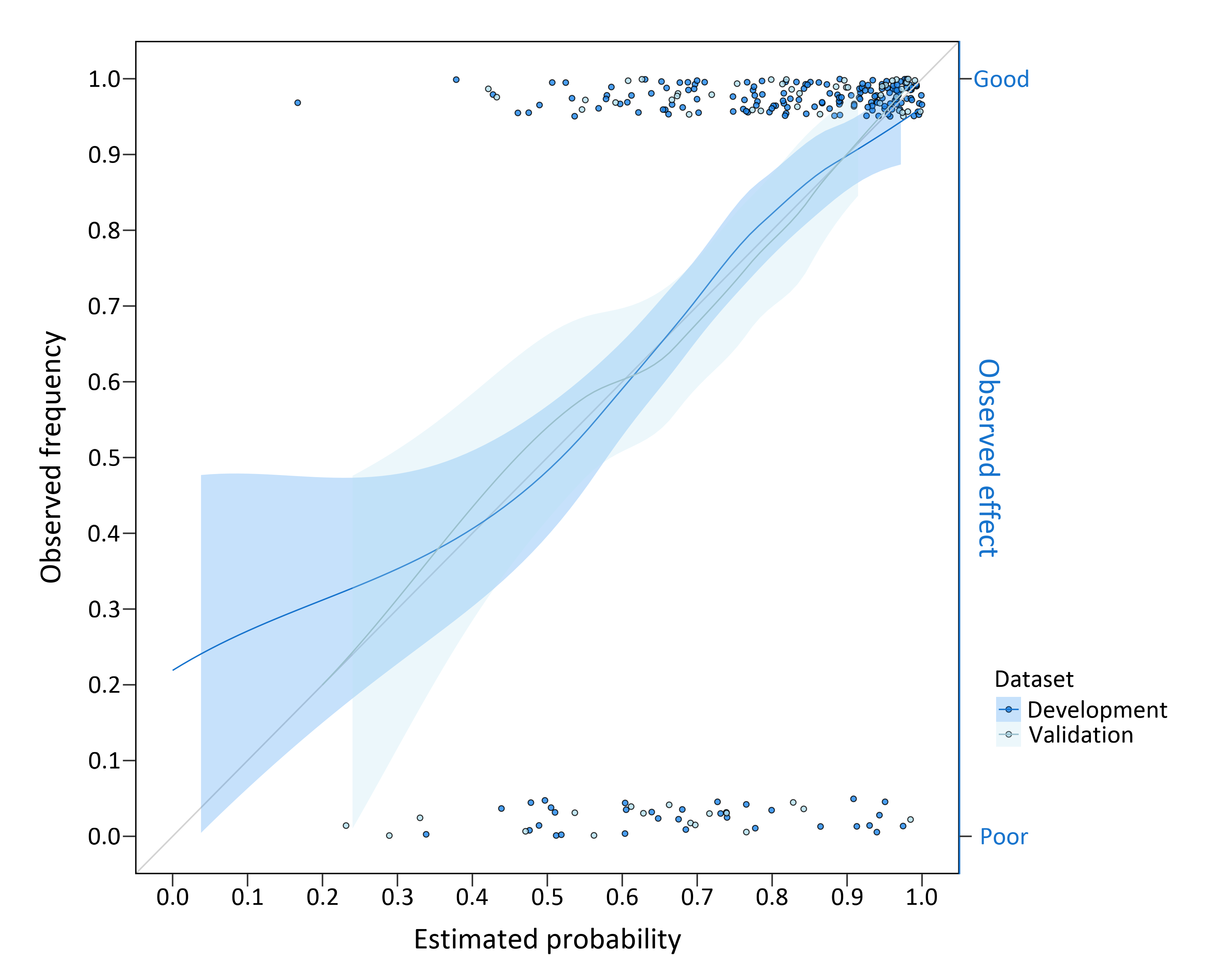
**

**
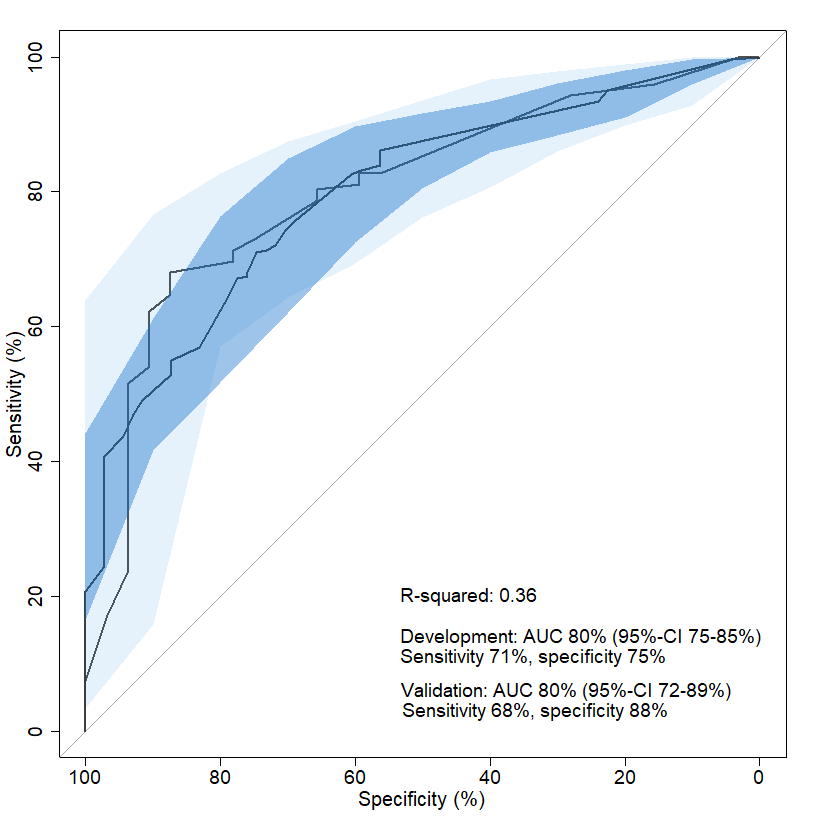
Supplementary figure 5: Performance of the original NEOS score in our cohort.**


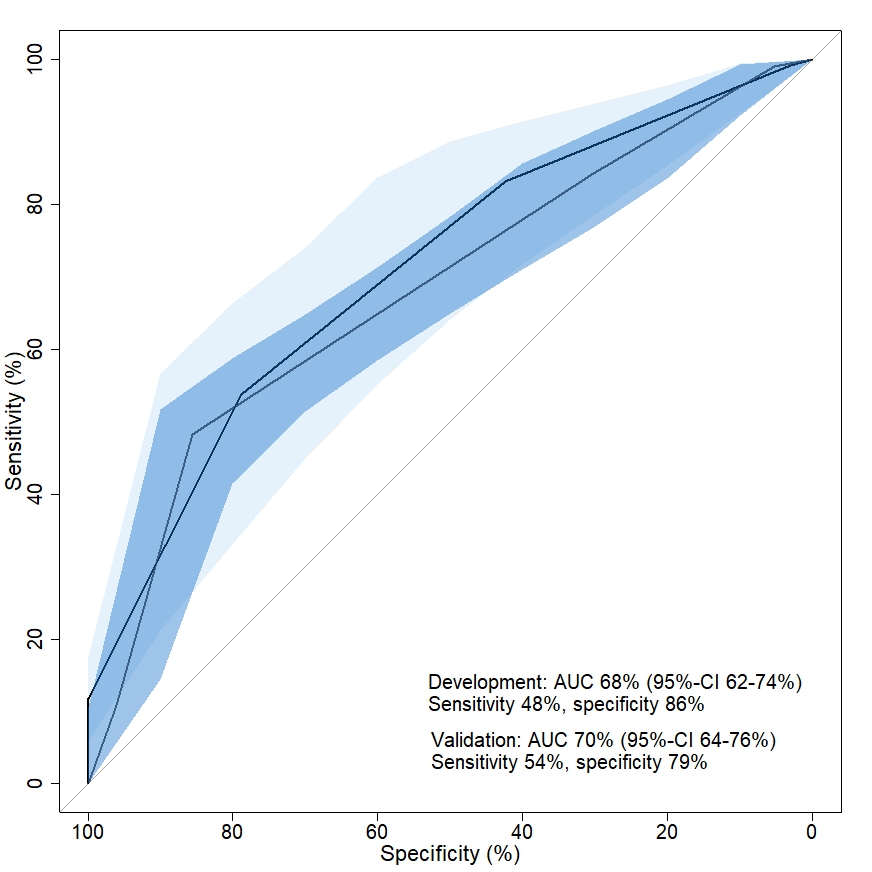
**Supplementary figure 6: Performance of a modified NEOS score, excluding treatment response in our cohort**

**Supplementary figure 7: Calibration plot of the multivariable model for early improvement after first-line treatment of anti-NMDAR encephalitis**.
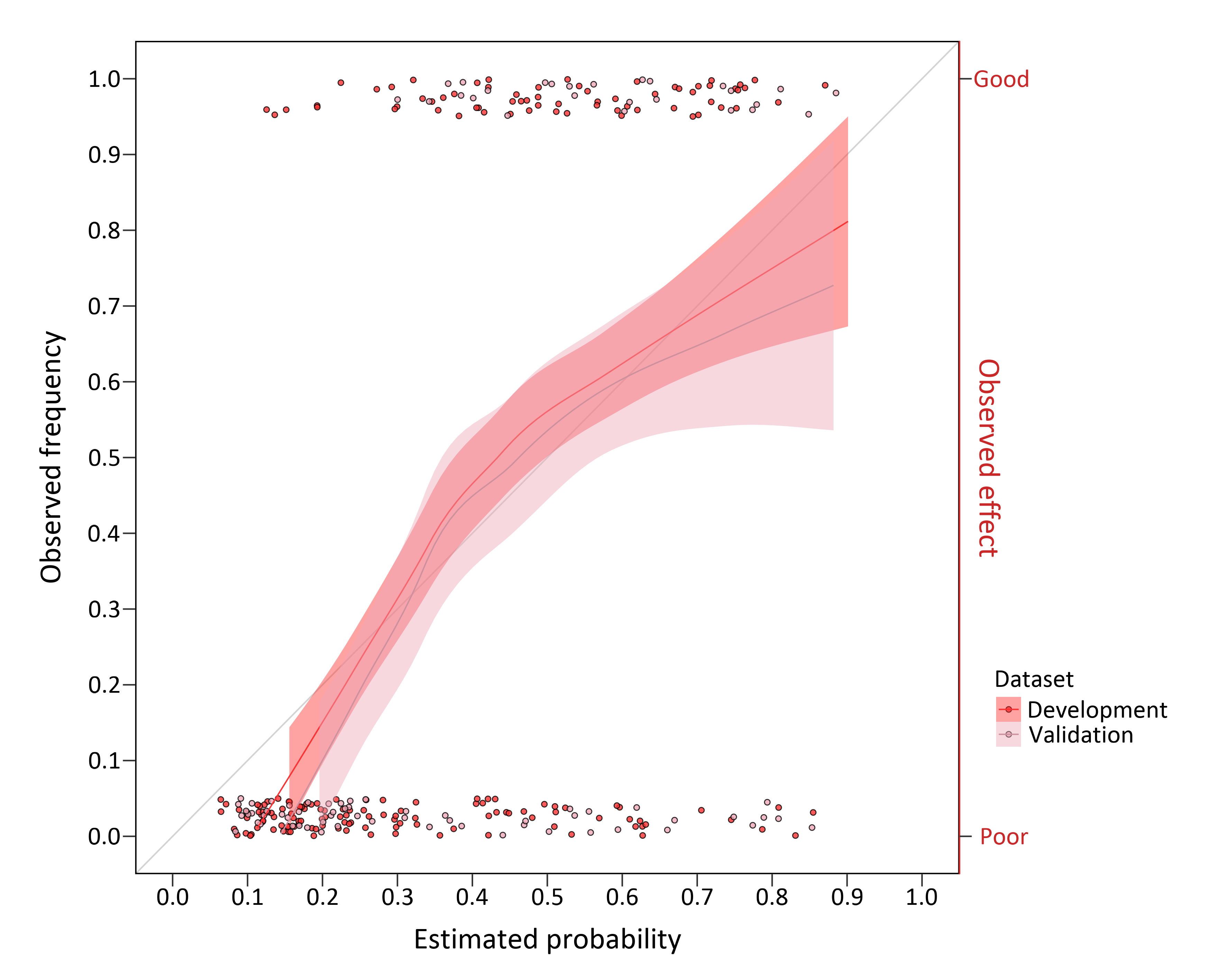


**Supplementary figure S8: Distribution of age at disease onset across cohorts.**



Distribution of age in the complete cohort and per country. The median age of the complete cohort was 22, the first 25% was under 16 years and 75% under 31.

**Figure S9: Distribution and directionality of treatment delay, and the relation to disease severity.**





a

b

c

d

A: Distribution of treatment delays in the cohort; quartiles were at 1½ weeks (25%), 2½ weeks (50%) and 5½ weeks (75%) and the distribution was positively skewed (figure is log-transformed). C & D: Visualizing the effect of treatment delay on early improvement after first-line therapy (C) and functional outcome after one year (D) revealed an unexpected non-linear effect of treatment delay on outcomes; a fast diagnosis and treatment was associated with a poorer prognosis, with inflection points around 2 (at 40% of the data), 4 (66%) and 8 weeks (80%). Adding treatment delay with splines to the multivariable model, demonstrated that this non-linear effect was largely explained by the other variables in the model reflecting disease severity. More severely affected patients were diagnosed and treated significantly faster (B; diagnostic delay mean 4 weeks, SD 3, when requiring ICU admission upon diagnosis vs. 5 weeks, SD 3, in patients not requiring ICU admission, p<0·0001; treatment delay mean 2, SD 3, vs 4, SD 3, p<0·0001; patients requiring ICU admission are represented by the dark-coloured dots in figure C & D). Besides the other variables in the multivariable model, a linear (negative) effect of (log-transformed) treatment delay remained.

**Figure S10: Distribution and transformation of CSF leukocyte number.**


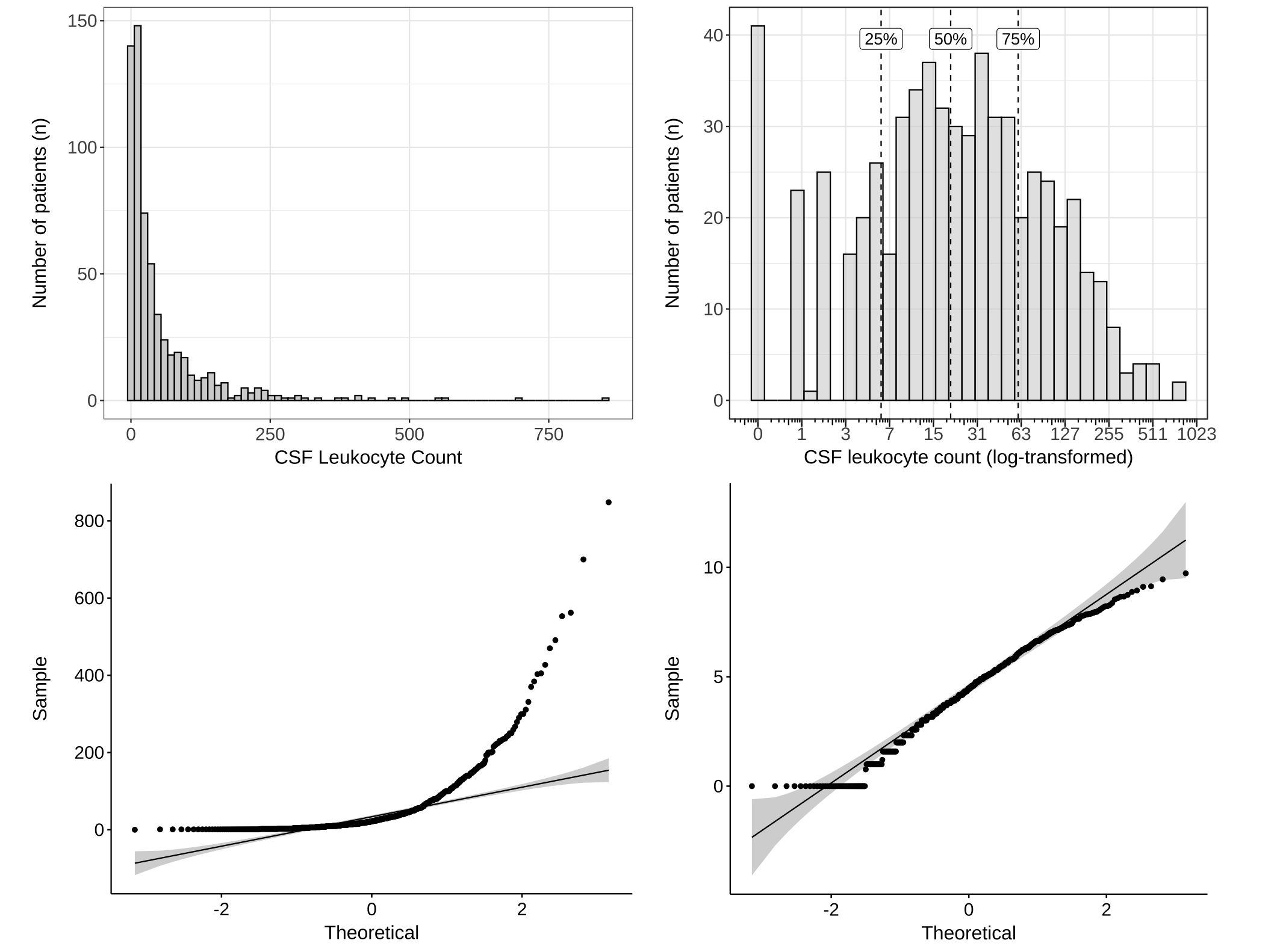


**Figure S11: Relapse risk was not easily predicted by the NEOS2-score.**


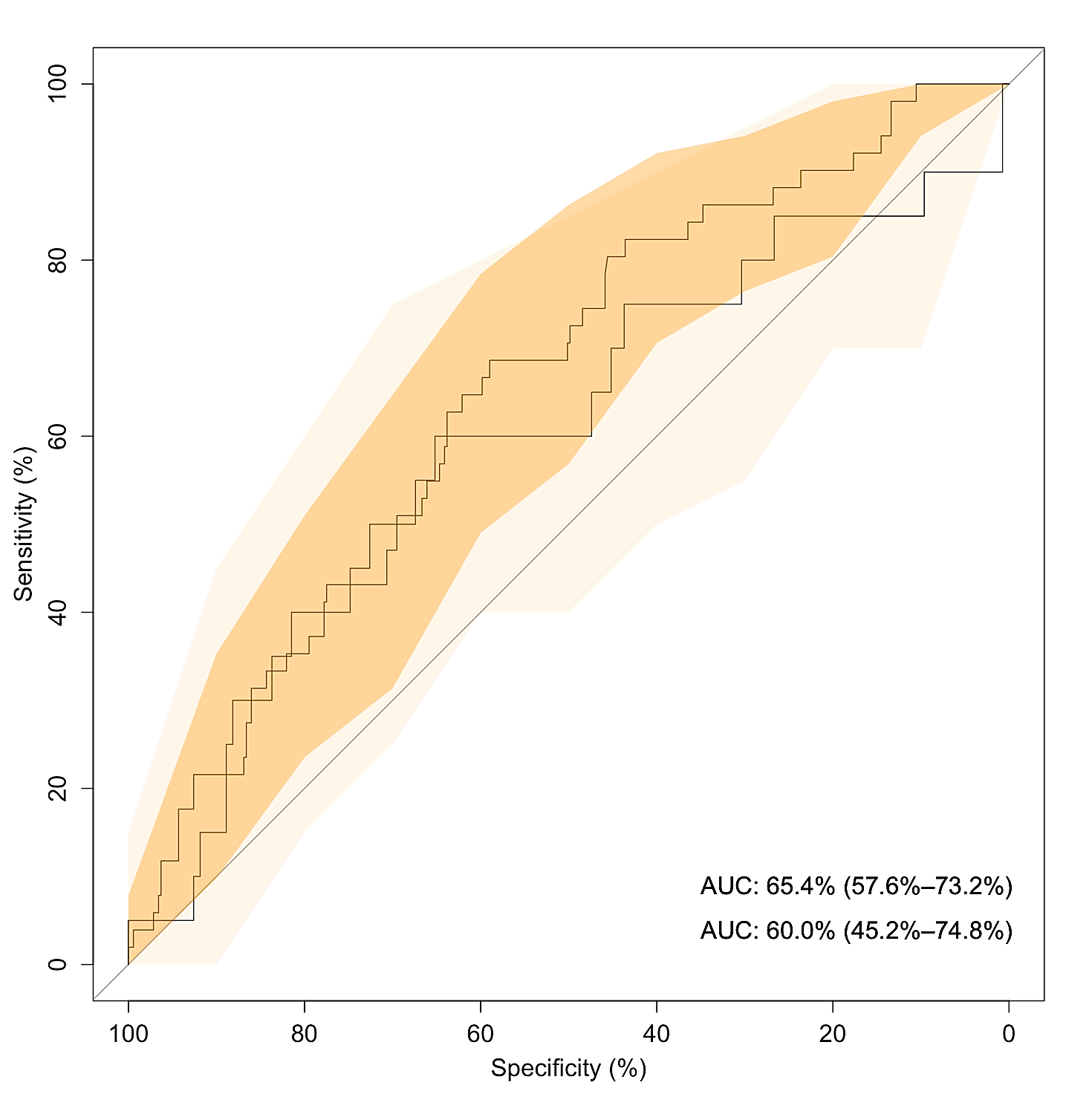


**SUPPLEMENTARY TEXT**

**Supplementary introduction**

In most prognostic studies, outcome is expressed in terms of independence (mRS≤2). As the majority of anti-NMDAR encephalitis patients reach functional independence after one (~80%) or two (>90%) years, outcomes are unevenly distributed, reducing the power of most prognostic studies. As a substantial part of anti-NMDAR encephalitis patients is not able to resume school or work at their premorbid level, even with favourable mRS scores,^1^ we expected this to be a more sensitive outcome measure for the longer term (beyond the first year), when most patients have returned to independence. We therefore introduce likelihood to return-to-work or -school as an outcome measure for the longer term, to be predicted (NEOS2-W) in addition to short-term (improvement after first-line therapy, within weeks; NEOS-T), and intermediate (improvement in the first year; NEOS2) outcomes.

Another way to increase the sensitivity of outcome measures, is to add the element of timing (within these first years); we did a time-to-event analysis to explore these additional prognostic factors.

**Supplementary method**

*Data transformation*

Continuous variables were normalized based on visualization, either with log2-transformation or taking the square root. We decided on log-transformation with 2 as a base, to increase interpretability of the odds ratios (OR); the OR of a log2-transformed variable conveys the change in odds when doubling the value of the predictive variable.

*Imputation*

Missing values on continuous variables were imputed by predictive mean matching additive regression, dichotomous variables with logistic regression.^2^ Missing values were imputed using the MICE package in R.^3^ All predictor variables and outcome values were used as predictive variables for the imputation, as well as extra information on recovery rates in the form of available mRS scores over time.

*Checking for linearity*

Effects of continuous variables were visualized and fitted with local polynomial regression curves to check for linearity. Age and treatment delay non-linearly related to long-term functional outcome in univariable analysis. Young adults under 36 years had the highest likelihood of a good functional outcome after one year (80-90%), children under the age of 12 a slightly lower one, and in adults over the age of 36 the likelihood exponentially decreased with age (Figure 5A). The exponential effect of the transformed variable motivated the inclusion of age as an untransformed variable with linear splines (<12, 12-36 and >36 years) in the multivariable model, to increase interpretability of the odds ratios.

More severely affected patients were diagnosed and treated significantly faster (diagnostic delay mean 4 weeks, SD 3, when requiring ICU admission upon diagnosis vs. 5 weeks, SD 3, in patients not requiring ICU admission, p<0·0001; treatment delay mean 2, SD 3, vs 4, SD 3, p<0·0001; Figure S9). A very fast diagnosis was therefore not more favourable then an average delay, although the odds of a good outcome decreased gradually beyond the average delay (OR 0·37, 95%-CI 0·16-0·78, p=0·013). This non-linear effect dissolved in multivariable analysis, when other variables reflecting disease severity were included, and a linear negative effect on outcome remained.

*Performance measures*

A way to assess performance of the multivariable models was to calculate the sensitivity and specificity for the outcome at different thresholds, plot receiver operating characteristic (ROC) curves and calculate the area under the curve (AUC) – accounting for both the sensitivity and specificity *across* thresholds. The AUC and 95% confidence intervals (CIs) for the AUC were calculated with the auc and ci.auc function from the pROC package in R. The ci.auc function employs a nonparametric, stratified bootstrap resampling approach to derive CIs.^e4^ The reported sensitivity and specificity are the optimal combination, at the threshold identified by the Youden’s statistic.^e5^ We would actually advise to apply the NEOS2-scores as ordinal scores to allow for a more nuanced weighing of the odds of achieving a good or poor outcome, and personalised clinical decision making, instead of imposing a threshold.

*International differences*

The cohorts from the Netherlands and France ensure national coverage, as the anti-NMDAR encephalitis diagnosis is always confirmed within the European accredited reference centre (ERN-RITA), presumingly providing an unbiased reflection of the disease population. The included Japanese patients were more severely affected at diagnosis, as only patients visiting a tertiary healthcare clinic were included. The Spanish cohort was part of a prospective study necessitating ability to perform extensive cognitive testing,^4^ explaining the high number of good outcomes at 12 months follow-up.

1. Brenner J, Ruhe CJ, Kulderij I, et al. Long-term cognitive, functional, and patient-reported outcomes in patients with anti-NMDAR encephalitis. Neurology 2024;103.

2. Rubin DB, Schenker N. Multiple Imputation for Interval Estimation From Simple Random Samples With Ignorable Nonresponse. Journal of the American Statistical Association 1986;81:366-374.

3. van Buuren S, Groothuis-Oudshoorn K. mice: Multivariate Imputation by Chained Equations in R. Journal of Statistical Software 2011;45:1 - 67.

4. Guasp M, Rosa-Justicia M, Munoz-Lopetegi A, et al. Clinical characterisation of patients in the post-acute stage of anti-NMDA receptor encephalitis: a prospective cohort study and comparison with patients with schizophrenia spectrum disorders. Lancet Neurol 2022;21:899-910.

**SUPPLEMENTARY REFERENCES**

e1. Van Buuren S, Groothuis-Oudshoorn K. MICE: Multivariate Imputation by Chained Equations in R. Journal of Statistical Software 2011;45:1-67.

e2. Team RC. R: A language and environment for statistical computing.: R Foundation for Statistical Computing, Vienna, Austria, 2023.

e3. Martijn W Heymans. psfmi: Prediction Model Pooling, Selection and Performance Evaluation Across Multiply Imputed Datasets 2021. R package version 1.1.0. <https://mwheymans.github.io/psfmi/>

e4. Robin X, Turck N, Hainard A, et al. pROC: an open-source package for R and S+ to analyze and compare ROC curves. BMC Bioinformatics 2011;12:77.

e5. Youden WJ. Index for rating diagnostic tests. Cancer 1950;3(1):32-35.

**AUTHOR CONTRIBUTIONS**

| **Name** | **Contributions** |
| --- | --- |
| J. Brenner | Conceptualisation, methodology, data acquisition, data curation, formal analysis, visualisation, writing and reviewing |
| A.E.M. Bastiaansen | Data acquisition, data curation, reviewing |
| M. Guasp | Data acquisition, data curation, reviewing |
| S. Muñiz-Castrillo | Data acquisition, data curation, reviewing |
| T. Iizuka | Data acquisition, data curation, reviewing |
| M.A.A.M. de Bruijn | Data acquisition, data curation, reviewing |
| A. Muñoz-Lopetegi | Data acquisition, data curation, reviewing |
| E. Martínez-Hernández | Data acquisition, data curation, reviewing |
| G. Picard | Data acquisition, data curation, reviewing |
| A. Vogrig | Data acquisition, data curation, reviewing |
| M. Millot | Data acquisition, data curation, reviewing |
| C. Finke | Data acquisition, data curation, reviewing |
| C. Geis | Data acquisition, data curation, reviewing |
| J. Lewerenz | Data acquisition, data curation, reviewing |
| N. Melzer | Data acquisition, data curation, reviewing |
| H. Prüss | Data acquisition, data curation, reviewing |
| S. Räuber | Data acquisition, data curation, reviewing |
| M. Ringelstein | Data acquisition, data curation, reviewing |
| K. Rostàsy | Data acquisition, data curation, reviewing |
| K.W. Sühs | Data acquisition, data curation, reviewing |
| F. Thaler | Data acquisition, data curation, reviewing |
| K.P. Wandinger | Data acquisition, data curation, reviewing |
| K. Wurdack | Data acquisition, data curation, reviewing |
| Y.S. Crijnen | Data acquisition, data curation, reviewing |
| J. Kerstens | Data acquisition, data curation, reviewing |
| R.W. van Steenhoven | Data acquisition, data curation, reviewing |
| S. Veenbergen | Resources, data acquisition, data curation, reviewing |
| M.W.J. Schreurs | Resources, data acquisition, data curation, reviewing |
| R. van den Berg | Methodology, reviewing |
| V. Volovici | Methodology, reviewing |
| R.F. Neuteboom | Data acquisition, reviewing |
| J.M. de Vries | Data acquisition, reviewing |
| P.A.E. Sillevis Smitt | Resources, project administration, reviewing |
| S.C. Franken | Data acquisition, data curation, reviewing |
| M.M.P. Nagtzaam | Data acquisition, data curation, reviewing |
| GENERATE study group | Data acquisition, data curation, reviewing |
| J. Dalmau | Data acquisition, data curation, reviewing |
| F. Leypoldt | Data acquisition, data curation, reviewing |
| J. Honnorat | Data acquisition, data curation, reviewing |
| M.J. Titulaer | Conceptualisation, methodology, funding acquisition, resources, project administration, supervision, validation, reviewing |

**GENERATE STUDY GROUP CONTRIBUTORS**

| **First Name** | **Surname** | **Affiliation** |
| --- | --- | --- |
| Dominica | Ratuszny | Department of Neurology, Hannover Medical School, Germany |
| Til | Menge | Department of Neurology, Center for Neurology and Neuropsychiatry, LVR-Klinikum, Heinrich-Heine-University Düsseldorf, Germany |
| Annikki | Bertolini | Department of Pediatric Neurology, Children's Hospital Witten/Herdecke University Datteln, Germany |
| Christian | Bien | Department of Neurology/Epilepsy, Hospital Mara Bethel, Germany |
| Robert | Berger | Department of Neurology, Asklepios Klinik Altona, Germany |
| Simone | Tauber | Department of Neurology, RWTH University Hospital Aachen, Germany |
| Klemens | Angstwurm | Department of Neurology, Medbo University Hospital Regensburg, Germany |
| Thomas | Seifert-Held | Department of Neurology, Medical University of Graz, Austria & Hospital Murtal Knittelfeld, Austria |
| Andrea | Kraft | Department of Neurology, Hospital Martha-Maria Halle, Germany |
| Jaqueline | Klausewitz | Department of Neurology, St. Josef Hospital, Ruhr-University Bochum, Germany |
| Ilya | Ayzenberg | Department of Neurology, St. Josef Hospital, Ruhr-University Bochum, Germany |
| Katharina | Eisenhut | Department of Neurology, University Hospital and Biomedical Center, Ludwig-Maximilians-Universität München, Germany |
| Rosa | Rößling | Department of Neurology, Charité Hospital Berlin, Germany |
| Martha | Heiden | Department of Neurology, Charité Hospital Berlin, Germany |
| Tania | Kümpfel | Department of Neurology, University Hospital and Biomedical Center, Ludwig-Maximilians-Universität München, Germany |
