# Supplementary material for: Development and validation of the NEOS2 score for prediction of long-term outcomes and improvement after first-line immunotherapy in patients with anti-NMDAR encephalitis: an international cohort study
Source: Lancet Reg Health Eur. 2025 Dec 11;62:101562. doi: 10.1016/j.lanepe.2025.101562 (PMC12757447; doi:10.1016/j.lanepe.2025.101562)
Supplement: Supplementary Material 2 [file mmc2.docx]

| **First Name** | **Surname** |
| --- | --- |
| Dominica | Ratuszny |
| Til | Menge |
| Annikki | Bertolini |
| Christian | Bien |
| Robert | Berger |
| Simone | Tauber |
| Klemens | Angstwurm |
| Thomas | Seifert-Held |
| Andrea | Kraft |
| Jaqueline | Klausewitz |
| Ilya | Ayzenberg |
| Katharina | Eisenhut |
| Rosa | Rößling |
| Martha | Heiden |
| Tania | Kümpfel |
